# Angiopoietin–TIE2 feedforward circuit promotes PIK3CA-driven venous malformations

**DOI:** 10.1038/s44161-025-00655-9

**Published:** 2025-05-23

**Authors:** Marle Kraft, Hans Schoofs, Milena Petkova, Jorge Andrade, Ana Rita Grosso, Rui Benedito, An-Katrien De Roo, Laurence M. Boon, Miikka Vikkula, Friedrich G. Kapp, René Hägerling, Michael Potente, Taija Mäkinen

**Affiliations:** 1 Uppsala University, Department of Immunology, Genetics and Pathology, Uppsala, Sweden; 2 Angiogenesis & Metabolism Laboratory, Center of Vascular Biomedicine, Berlin Institute of Health at Charité – Universitätsmedizin Berlin, Berlin, Germany; 3 Max Delbrück Center for Molecular Medicine in the Helmholtz Association, Berlin, Germany; 4 Associate Laboratory i4HB - Institute for Health and Bioeconomy, NOVA School of Science and Technology, Universidade NOVA de Lisboa, Lisbon, Portugal; 5 UCIBIO – Applied Molecular Biosciences Unit, Department of Life Sciences, NOVA School of Science and Technology, Universidade NOVA de Lisboa, Lisbon, Portugal; 6 Molecular Genetics of Angiogenesis Group. Centro Nacional de Investigaciones Cardiovasculares (CNIC), Madrid, Spain; 7 Center for Vascular Anomalies, VASCERN VASCA European Reference Center, Cliniques Universitaires Saint Luc, UCLouvain, Brussels, Belgium; 8 Department of Pathology, Cliniques Universitaires Saint-Luc, Université Catholique de Louvain (UCLouvain), Brussels, Belgium; 9 Institute of Experimental and Clinical Research, UCLouvain, Brussels, Belgium; 10 Division of Plastic Surgery, Cliniques Universitaires Saint-Luc, Université Catholique de Louvain (UCLouvain), Brussels, Belgium; 11 Laboratory of Human Molecular Genetics, de Duve Institute, UCLouvain, Brussels, Belgium; 12 WELBIO Department, WEL Research Institute, Wavre, Belgium; 13 Department of Pediatric Hematology and Oncology, Children’s Hospital, Medical Center - University of Freiburg, Faculty of Medicine, University of Freiburg, VASCERN VASCA European Reference Center, Freiburg, Germany; 14 Research Group ‘Lymphovascular Medicine and Translational 3D-Histopathology’, Institute of Medical and Human Genetics, Charité-Universitätsmedizin Berlin, Berlin, Germany; 15 Berlin Institute of Health at Charité-Universitätsmedizin Berlin, BIH Center for Regenerative Therapies, Berlin, Germany; 16 Translational Cancer Medicine Program and Department of Biochemistry and Developmental Biology, University of Helsinki, Helsinki, Finland; 17 Wihuri Research Institute, Helsinki, Finland

**Keywords:** Vascular diseases, Cells, Preclinical research, Vascular diseases

## Abstract

Venous malformations (VMs) are vascular anomalies lacking curative treatments, often caused by somatic *PIK3CA* mutations that hyperactivate the PI3Kα–AKT–mTOR signaling pathway. Here, we identify a venous-specific signaling circuit driving disease progression, where excessive PI3Kα activity amplifies upstream TIE2 receptor signaling through autocrine and paracrine mechanisms. In *Pik3ca*^*H1047R*^-driven VM mouse models, single-cell transcriptomics and lineage tracking revealed clonal expansion of mutant endothelial cells with a post-capillary venous phenotype, characterized by suppression of the AKT-inhibited FOXO1 and its target genes, including the TIE2 antagonist ANGPT2. An imbalance in TIE2 ligands, likely exacerbated by aberrant recruitment of smooth muscle cells producing the agonist ANGPT1, increased TIE2 activity in both mouse and human VMs. While mTOR blockade had limited effects on advanced VMs in mice, inhibiting TIE2 or ANGPT effectively suppressed their growth. These findings uncover a PI3K–FOXO1–ANGPT–TIE2 circuit as a core driver of PIK3CA-related VMs and highlight TIE2 as a promising therapeutic target.

## Abstract (web summary)

Kraft et al. identify TIE2 activation as a key driver of *PIK3CA*-related venous malformations and demonstrate that inhibiting TIE2 signaling can suppress the growth of advanced lesions.

## Main

VMs are a chronic disease of the blood vasculature, characterized by localized abnormalities encompassing a spectrum of presentations from superficial, asymptomatic blue or purplish marks to potentially life-threatening lesions. VMs may develop in various tissues, with a high prevalence in the skin and subcutaneous tissue^1^. Classified as ‘slow-flow’ vascular anomalies, they are characterized by enlarged veins with abnormal smooth muscle cell (SMC) coverage and disrupted blood flow, and are often associated with increased platelet aggregation, coagulation, hemorrhages and swelling^1^.

Most VMs arise from somatic mutations that occur early during development, disrupting normal vessel formation processes. The most common among these are activating mutations in genes encoding the endothelial TIE2 receptor tyrosine kinase (encoded by *TEK*)^2^, or its downstream effector, the lipid kinase PI3Kα (encoded by *PIK3CA*)^2–4^. The TIE2–PI3K signaling pathway plays a pivotal role in blood vessel remodeling during development^5,6^. TIE2 signaling is also involved in the establishment of venous endothelial identity^7^, as well as the maintenance of vascular stability and homeostasis in adult tissues^6,8^. These diverse effects are finely balanced by two key TIE2 ligands, angiopoietin 1 (ANGPT1) and angiopoietin 2 (ANGPT2). ANGPT1 is produced by perivascular cells and acts in a paracrine manner as a TIE2 agonist, promoting vascular stability. ANGPT2, on the other hand, is produced by endothelial cells (ECs) themselves and primarily functions as an antagonist that destabilizes blood vessels^6,9^. In line with this, increased ANGPT2 levels have been associated with blood vessel instability and leakage, contributing to vascular dysfunction in conditions such as sepsis and inflammation^10^, while ANGPT1 prevents blood vessel leakage^8^. Notably, the effects of ANGPT2 are highly context dependent, as in certain situations it can also act as a weak agonist^8^. ANGPT2 can also signal through integrins independently of TIE2 (ref. ^11^) and can promote TIE2 interaction with the closely related TIE1 receptor^12^.

Individuals with VMs caused by *TEK* or *PIK3CA* mutations have shown promising responses to rapamycin (sirolimus), which targets mTOR—a downstream component of the PI3K–AKT pathway^1^. More recently, alpelisib, a specific PI3Kα inhibitor, has been used to treat PIK3CA-related overgrowth spectrum, often including VMs^13^. While these treatments alleviate symptoms, they are rarely curative and have limited impact on established lesions^1^. Studies in patient-derived ECs and in genetic mouse models have demonstrated the expected increase in cell proliferation upon PI3K–AKT activation^3,4,14–16^. However, this effect seems restricted to early-stage lesions, as established lesions in both humans^17^ and mice^16^ are non-proliferative. Mouse models of *Pik3ca*-related overgrowth have further underscored the exquisite sensitivity of ECs to abnormal PI3K signaling^3,4^ and the necessity of active angiogenesis for lesion development in the retina and the central nervous system^15,18^. Despite these insights, questions remain regarding the mechanisms that drive disease progression and determine the predominant manifestation in skin and subcutaneous tissues.

Here, we used a genetic mouse model of VMs in combination with single-cell transcriptomics to study mechanisms underlying *Pik3ca*-driven venous-specific vascular overgrowth. Our findings reveal an autocrine feedforward mechanism whereby aberrant PI3Kα activation amplifies TIE2 activity due to an imbalance in the levels of the TIE2 ligands. This imbalance is partly caused by AKT-mediated inhibition of FOXO1, leading to reduced transcription of the antagonistic ligand ANGPT2, concomitant with the recruitment of SMCs producing the agonistic ligand ANGPT1. Importantly, neutralizing these ligands with a soluble TIE2 protein, or inhibiting TIE2 pharmacologically, reduced *Pik3ca*-driven VM growth in mice, highlighting a promising therapeutic strategy for VM treatment.

## Results

### *Pik3ca*-driven VMs form in skin independently of angiogenesis

**Fig. 1:**
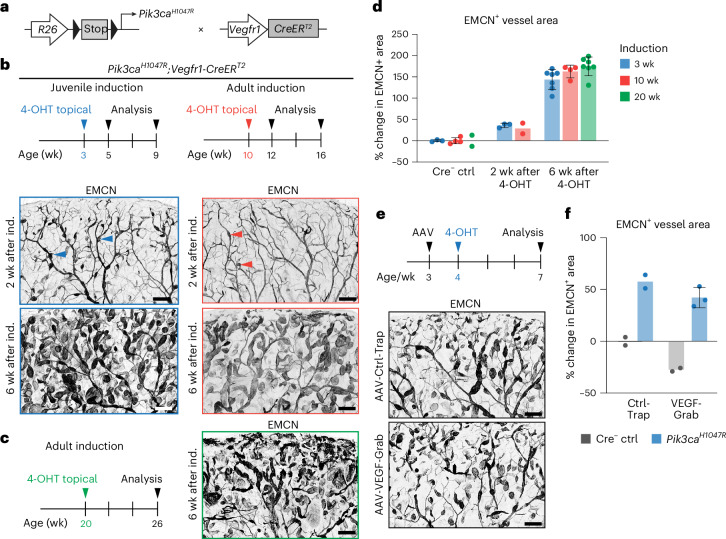
Formation of *Pik3ca*-driven cutaneous VMs in the absence of developmental angiogenesis and VEGF. **a**, Genetic constructs for tamoxifen-inducible BEC-specific expression of *Pik3ca*^*H1047R*^. **b**,**c**, Experimental scheme for *Pik3ca*-driven VMs in ear skin following 4-OHT induction at 3 weeks, 10 weeks (**b**) or 20 weeks (**c**) of age (50 μg, topical application to each ear) and analysis. Images show whole-mount immunofluorescence of ear skin with VM lesions (arrowheads) from 4-OHT-treated *Pik3ca*^*H1047R*^*;Vegfr1-CreER*^*T2*^ mice at the indicated time points. **d**, Quantification of vessel growth 2 weeks and 6 weeks after 4-OHT induction. Data represent an increase in EMCN^+^ vessel area relative to Cre^−^ littermate control (Ctrl), mean ± s.d. (sample size at 3, 10 and 20 weeks: Ctrl: *n* = 3, 4 and 2 mice, respectively; 2 weeks after 4-OHT, *n* = 3, 2 and NA mice, respectively; 6 weeks after 4-OHT, *n* = 7, 4 and 7 mice, respectively). **e**, Experimental scheme (top) for the inhibition of VEGF signaling by intraperitoneal injection of AAV vectors encoding VEGF-Grab or a control molecule before induction of vessel overgrowth in the ear skin of *Pik3ca*^*H1047R*^*;Vegfr1-CreER*^*T2*^ mice, visualized below by whole-mount immunofluorescence. **f**, Quantification of vessel growth, shown as a change in EMCN^+^ vessel area relative to Cre^−^ littermate Ctrl, mean ± s.d. (*n* = 2 (Ctrl + Ctrl-Trap), *n* = 2 (*Pik3ca*^*H1047R* ^+ Ctrl-Trap), *n* = 2 (Ctrl + VEGF-Grab), *n* = 3 (*Pik3ca*^*H1047R*^ + VEGF-Grab) mice). Scale bars, 100 μm (**b**, **c** and **e**). Source data

**Extended Data Fig. 1:**
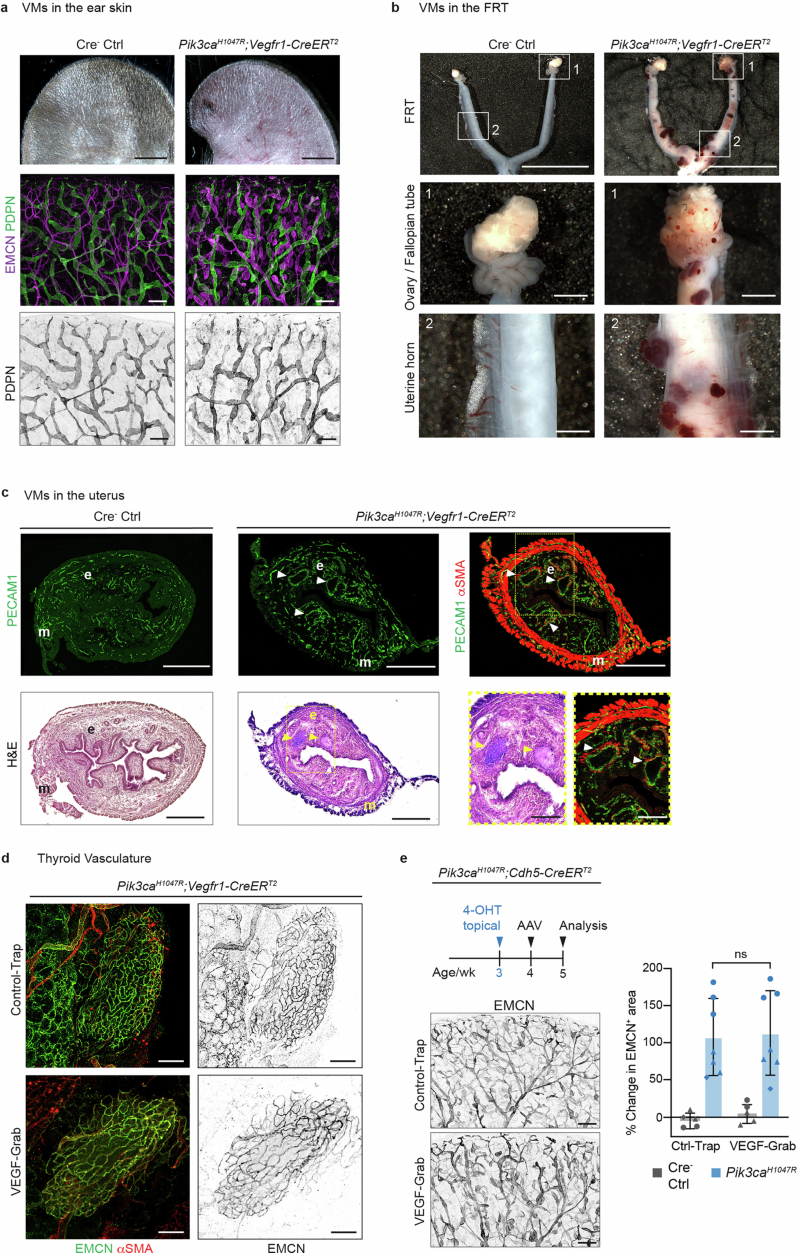
Characterization of *Pik3ca*-driven VM-formation across tissues and their dependency on VEGF. (**a**) Top: Bright field images of ears from 4-OHT-treated *Pik3ca*^*H1047R*^;*Vegfr1-CreER*^*T2*^ mice and Cre^-^ littermate control mice (Ctrl) four weeks post-induction, showing macroscopic VM lesions and redness of ears skin in the mutant. Below: Whole-mount immunofluorescence showing overgrowth of EMCN^+^ veins but unaffected PDPN^+^ lymphatic vessels in the mutant. Similar results were obtained from 7 mice in 2 independent experiments. (**b**) Female reproductive tract (FRT) following topical application of 4-OHT (50 μg) to the ear skin of 3-week-old *Pik3ca*^*H1047R*^;*Vegfr1-CreER*^*T2*^ mice and Cre^-^ littermate control mice (Ctrl), showing vascular lesions in ovaries, fallopian tube and the uterine horn in the mutant. Boxed regions are magnified below. Similar results were obtained from 10 mice in 6 independent experiments. (**c**) Immunofluorescence (top) and Hematoxylin and Eosin staining (H&E) (below) of cryo-sections visualizing ECs (PECAM1) and SMCs (αSMA) in the uterus of *Pik3ca*^*H1047R*^;*Vegfr1-CreER*^*T2*^ and Cre^-^ littermate control mice (Ctrl). Arrowheads point to enlarged, SMC-covered vasculature in endometrial stroma (e). The boxed regions in both the H&E and immunofluorescence images correspond to the same areas, which are magnified on the right. m, myometrium. Similar results were obtained from 4 mice in 2 independent experiments. (**d**) Thyroid vasculature four weeks after intraperitoneal injection of AAV vectors encoding VEGF-Grab or a control molecule, and three weeks post-4-OHT induction. EMCN^+^ veins and αSMA^+^ SMCs are visualized. Similar results were obtained from 3 mice in 1 experiment. (**e**) Top: Experimental scheme for the induction of VMs followed by intraperitoneal injection of AAV vectors encoding VEGF-Grab or a control molecule to inhibit VEGF signaling. Below: Whole-mount immunofluorescence images of ear skin from VEGF-Grab or control-Trap-treated *Pik3ca*^*H1047R*^;*Cdh5-CreER*^*T2*^ mice. Right: Quantification of vessel growth, represented as a change in EMCN^+^ vessel area relative to Cre^-^ littermate control (Ctrl), mean ± sd (n = 4 (Ctrl+Ctrl-Trap), n = 7 (*Pik3ca*^*H1047R*^+Ctrl-Trap), n = 6 (Ctrl+VEGF-Grab), n = 7 (*Pik3ca*^*H1047R*^ + VEGF-Grab) mice) from three independent experiments, indicated by symbol. ns, P = 0.8562 (not significant, ns), Unpaired two-tailed Student’s t-test. Scale bars: 1 cm (**a**, overview), 1 mm (**a**, **b** magnification), 500 μm (**c**, low magnification), 200 μm (**d**, **c** high magnification). Source data

We employed a mouse model of *Pik3ca*^*H1047R*^-driven VMs, using the tamoxifen-inducible blood endothelial cell (BEC)-specific *Vegfr1-CreER*^*T2*^ deleter^16^, to investigate the mechanisms controlling VM progression (Fig. 1a). Consistent with our previous findings^16^, expression of the causative *Pik3ca*^*H1047R*^ mutation in the ear skin vasculature of 3-week-old juvenile mice resulted in blood-filled vascular lesions (Extended Data Fig. 1a), which progressively grew within endomucin (EMCN)-positive veins and capillaries (Fig. 1b), while lymphatic vessels were unaffected (Extended Data Fig. 1a). Cre^−^ littermate mice treated with 4-hydroxytamoxifen (4-OHT) and carrying the *Pik3ca*^*H1047R*^ allele, used as controls, were also unaffected (Extended Data Fig. 1a).

Previous studies in the mouse retina have reported that *Pik3ca*-driven VM growth requires active angiogenesis^15^. To determine if cutaneous VMs show a similar dependence, we induced *Pik3ca*^*H1047R*^ expression at 10 or 20 weeks of age when the dermal vasculature is no longer proliferating^16^. Unexpectedly, *Pik3ca*^*H1047R*^ still promoted vascular lesion formation in this quiescent dermal vasculature (Fig. 1b–d). Two weeks after topical application of 4-OHT to the adult ear skin, vascular lesions were smaller compared to those induced in juvenile mice (Fig. 1b,d). However, by 6 weeks after induction, the overgrowth and phenotypic characteristics were comparable across the three induction protocols (Fig. 1b–d). Although systemic low-frequency recombination was also observed in other vascular beds with this treatment regime^16^, VM lesions consistently appeared only in select organs, such as the female reproductive organs (Extended Data Fig. 1b,c). The susceptibility of BECs in the uterus, fallopian tube and ovaries to *Pik3ca*^*H1047R*^-driven overgrowth (Extended Data Fig. 1b,c) likely reflects the continuous angiogenic state of these tissues.

To investigate the dependence of cutaneous VMs on the key angiogenic regulator vascular endothelial growth factor (VEGF), *Pik3ca*^*H1047R*^*;Vegfr1-CreER*^*T2*^ mice were treated one week before 4-OHT induction with an adeno-associated virus (AAV) encoding a soluble VEGF-Grab, which neutralizes VEGF-A, VEGF-B and placenta growth factor^19^. An AAV encoding a control trap, incapable of ligand binding, was used as a control (Fig. 1e). The effectiveness of VEGF neutralization was validated by the VEGF-Grab-induced reduction of the thyroid vasculature (Extended Data Fig. 1d), a vascular bed known to be highly sensitive to VEGF blockade^20^. While VEGF neutralization in juvenile mice reduced EMCN^+^ dermal vessel area in control mice, it failed to inhibit lesion formation in *Pik3ca*^*H1047R*^ mice (Fig. 1e,f). Neutralizing VEGF one week after the onset of VM lesion formation also did not affect lesion growth (Extended Data Fig. 1e), suggesting that VEGF signaling is not required for VM formation and progression. Taken together, these findings demonstrate susceptibility of the mouse dermal vasculature to form *Pik3ca*^*H1047R*^-driven vascular lesions, a vascular bed often affected in individuals with VM carrying this mutation^1^. This susceptibility persists beyond the developmental period and even in the absence of paracrine VEGF.

### Single-cell transcriptomics of mouse skin microvasculature

**Fig. 2:**
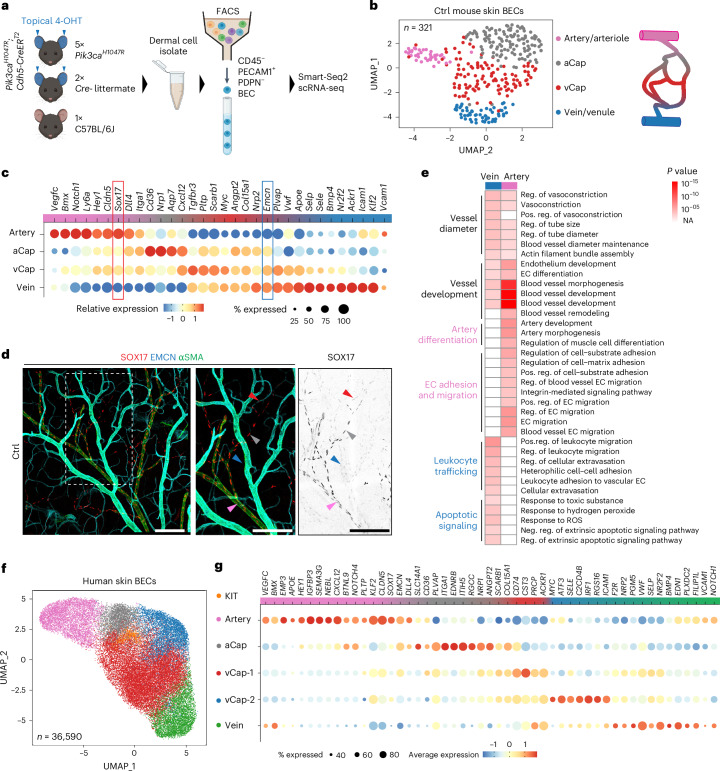
AV zonation and post-capillary venous characteristics of the dermal microvasculature. **a**, Experimental scheme for Smart-Seq2 scRNA-seq analysis of dermal BECs from 5-week-old *Pik3ca*^*H1047R*^*; Cdh5-CreER*^*T2*^ (*n* = 5) and Ctrl (*n* = 3) mice 2 weeks after topical application of 25 µg of 4-OHT to each ear. **b**, UMAP representation of 321 dermal BECs from Ctrl mice. Four BEC clusters after Harmony batch effect correction are annotated and schematically matched to their anatomic position within the vascular bed on the right (color coded according to UMAP). **c**, Dot plot of markers defining the four subclusters of BECs from **b**. Exemplary arterial (*Sox17*) and venous (*Emcn*) markers are highlighted. **d**, Whole-mount immunofluorescence of ear skin showing EMCN and SOX17 expression across the AV axis in a Ctrl mouse. Antibody against αSMA was used to visualize SMC coverage in arteries. Arrowheads are color coded according to UMAP in **b**, indicating vessel type. Similar results were obtained from two mice. Scale bar, 200 µm. **e**, GO enrichment analysis applying GOstats to the top vein and artery cluster markers using a standard hypergeometric test with a significance threshold of *P* < 0.00001). *P* values of GO terms are encoded by color gradient; NA indicates no enrichment. Groups of cluster-specific terms are color coded accordingly. **f**, UMAP representation and annotation of 36,590 dermal BECs from 23 healthy human individuals after Seurat’s anchor-based integration. **g**, Dot plot of relative marker expression defining the six subclusters of BECs from **f**. Dot sizes in **c** and **g** represent transcript percentage in each cluster. Color illustrates the average expression compared across displayed clusters. FACS, fluorescence-activated cell sorting. NA, not applicable. Panel **a** created with BioRender.com. Source data

To characterize the BEC-autonomous mechanisms involved in VM formation, we first defined the transcriptome of the normal dermal microvasculature, to allow comparison to *Pik3ca* mutant mice. To this end, we performed Smart-Seq2 single-cell RNA sequencing (scRNA-seq) of dermal BECs isolated by flow cytometry based on selection of PECAM1^+^PDPN^−^CD45^−^ cells from the ears of 4-OHT-treated *Pik3ca*^*H1047R*^*;Cdh5-CreER*^*T2*^ and Cre^−^ littermate mice, as well as an untreated wild-type C57BL/6J mouse^16^ (Fig. 2a). Previous comparisons of *Cdh5-CreER*^*T2*^-driven and *Vegfr1-CreER*^*T2*^-driven VM models showed similar phenotypes across several parameters^16^. In total, 1,595 quality-controlled cells were processed using the Seurat v3 workflow with default settings for normalization, feature selection, linear transformation and dimensional reduction. During the analysis, 145 cells expressing markers of keratinocytes (*Krt16*, *Krt42*), platelets (*Gp1ba*, *Pf4*), SMCs (*Acta2*, *Pdgfrb*) or fibroblasts (*Lum*, *Col1a2*) were identified and removed as contaminants.

**Extended Data Fig. 2:**
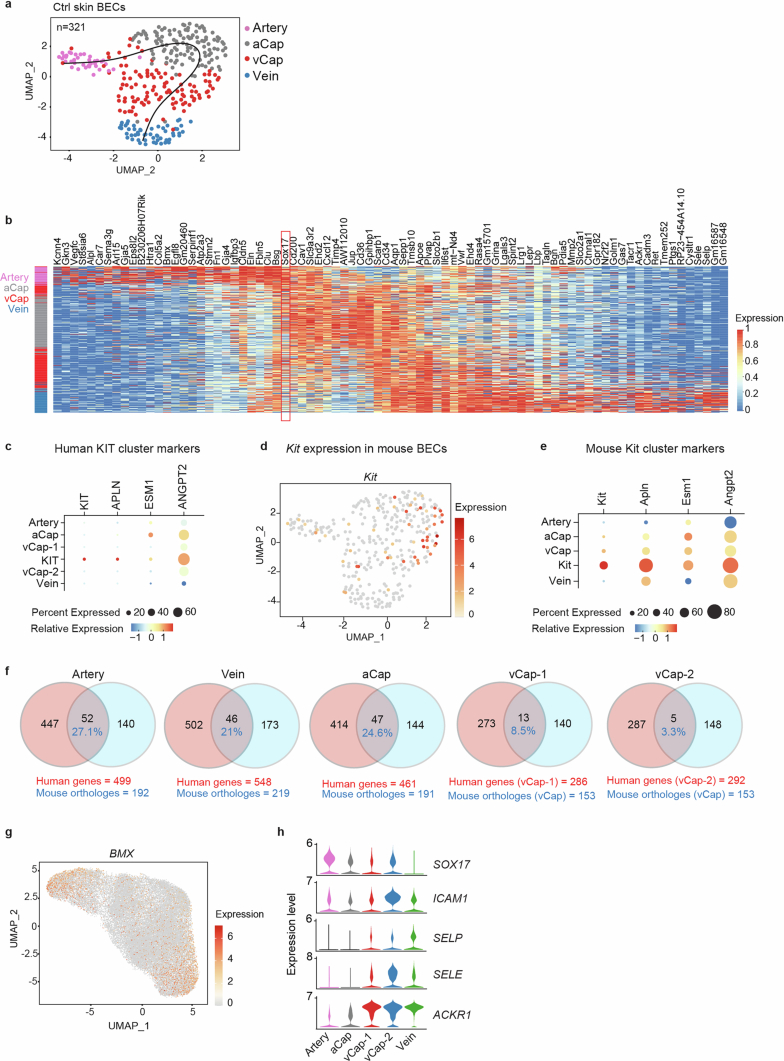
Trajectory and cross-species conservation analysis of dermal BECs. (**a**) Unsupervised trajectory inference analysis of dermal BECs from control mice using SCORPIUS, visualized in a UMAP plot. Black line indicates the trajectory. (**b**) Heatmap of zonation marker expression across cells ordered by cluster and along their trajectory, according to UMAP representation in **a**. (**c**) Dot plot of top genes characterizing KIT cluster in human BECs. (**d**) UMAP-embedded representation of *Kit* expression levels in control mouse BECs. (**e**) Dot plot of top genes characterizing Kit cluster in integrated control and *Pik3ca* mouse BECs. Dot size in **c** and **d** represents transcript percentage in each cluster, color illustrates the average expression compared across displayed clusters. (**f**) Venn diagram representation of conservation analysis of gene expression between mouse and human BEC clusters, based on the UMAP representations in Fig. 2b, f. (**g**) UMAP-embedded representation of *BMX* expression levels in human BECs, marking larger vessels at the terminal ends of arterial and venous clusters. (**h**) Violine plots of immune-associated post-capillary venule markers, and capillary marker *SOX17*, in human BECs, according to UMAP representation in Fig. 2f. Source data

We extracted 321 BECs from control mice and conducted batch effect correction using Seurat canonical correlation analysis. Subsequent analysis revealed four distinct clusters based on the expression of established EC-identity markers: arterial (for example, *Vegfc*, *Bmx* and *Hey1*) and venous (for example, *Nrp2*, *Emcn* and *Nr2f2* (encoding COUP-TFII)) ECs, as well as two distinct capillary (Cap) EC populations (Fig. 2b,c). Unsupervised trajectory inference analysis using SCORPIUS demonstrated phenotypic zonation along the arteriovenous (AV) axis and across the two capillary subpopulations, termed post-arterial capillaries (aCap) and pre-venule capillaries (vCap; Extended Data Fig. 2a,b). The aCap and vCap clusters showed extensive overlap in marker expression, with aCap resembling arterial ECs and vCap resembling venous ECs. For example, a decrease in *Sox17* expression was observed along the AV axis, which coincided with a concomitant increase in *Emcn* levels (Fig. 2c and Extended Data Fig. 2b). Additional cluster markers and data are available at https://makinenlab.shinyapps.io/Mouse_DermalBloodEndothelialCells/. Whole-mount immunofluorescence of the ear skin confirmed high SOX17 expression in arteries and extending into capillaries but absent in regions with high EMCN expression (Fig. 2d). This zonated expression pattern thus allowed the distinction between *Sox17*-expressing arterial and aCap ECs (*Sox17*^high^*Emcn*^−^) from vCap ECs (*Sox17*^low^*Emcn*^low^) and venous ECs (*Sox17*^−^*Emcn*^high^).

Notably, the venous cluster was characterized by high expression of genes encoding regulators of immune cell rolling and diapedesis (*Selp*, *Sele*, *Ackr1*, *Icam1*; Fig. 2c), characteristic of post-capillary venules that are specialized for immune cell trafficking^21,22^. Gene Ontology (GO) analysis of cluster markers further revealed enrichment of biological processes associated with immune cell trafficking in the venous cluster, while the arterial cluster was characterized by processes related to EC migration, arterial fate decision and EC communication with the extracellular matrix and SMCs (Fig. 2e).

### Conserved transcriptional zonation of human skin vasculature

To investigate the conservation of these signatures in humans, we extracted expression data from five publicly available scRNA-seq datasets of human skin (Methods). In total, 45,131 BECs were initially selected based on the coexpression of pan-EC markers (*CD31*, *CDH5*) and the BEC-specific marker *FLT1*, while lymphatic ECs expressing *PROX1* were excluded. After quality control and removal of contaminating cells expressing SMC markers (for example, *PDGFRB*, *ACTA2*), 36,590 BECs from 23 healthy individuals were included for further analysis. After correcting for batch effects and technical variation, BECs were distributed into six clusters (Fig. 2f), defined as arterial (for example, *VEGFC*, *HEY1*), venous (for example, *NRP2*, *NR2F2*) and three capillary (for example, *COL15A1*, *PLVAP*) EC populations (Fig. 2g). These capillary EC populations were further classified into arterial (aCap) and venous identities (vCap1, vCap2), with the latter distinguished by the expression of *ATF3*, previously described in the skeletal muscle and lung Cap ECs^23,24^, as well as genes encoding regulators of cellular stress (*FOS*, *JUN*; data not shown), growth and metabolism (*MYC*) and immune cell trafficking (*ICAM1*, *SELE*; Fig. 2g).

A distinct cluster was observed at the interface between aCap and vCap BECs in the uniform manifold approximation and projection (UMAP) representation, expressing *KIT* and the angiogenic tip cell markers *ESM1* and *APLN* (Fig. 2g and Extended Data Fig. 2c)^25^. A comparable population was identified in the mouse dataset in a similar UMAP position, although it did not form a separate cluster due to low cell numbers (Extended Data Fig. 2d,e). Although human arterial, venous and capillary clusters broadly displayed shared marker expressions similar to those identified in mouse BECs (Fig. 2g; https://makinenlab.shinyapps.io/Human_DermalBloodEndothelialCells/) and previous analyses of human vasculature^26^, some exceptions were observed. For instance, the expression of mouse venous EC markers (for example, *EMCN*, *KLF2*, *APOE*), which were present in human arterial but not venous ECs (Fig. 2g). In contrast, SOX17 was expressed in arterial and capillary ECs (Fig. 2g and Extended Data Fig. 2h), as observed in mice. Despite the overall high overlap of artery, vein and aCap markers, lower conservation was observed for the venous capillary populations vCap1 and vCap2 (Extended Data Fig. 2f). Additionally, we noted shared markers in arterial and venous ECs at the terminal end of the clusters, suggesting zonation toward larger vessel types (for example, *BMX;* Fig. 2g and Extended Data Fig. 2g), *FBLN2* and *LTBP4* (data not shown). Notably, *ANGPT2* was expressed among all human capillary subpopulations, but its expression was less prevalent in veins and absent in *BMX*-expressing ECs of larger arteries and veins (Fig. 2g and Extended Data Fig. 2c). Human dermal venous and vCap BECs also exhibited high expression of post-capillary venule genes, including *ICAM1*, *SELP*, *SELE* and *ACKR1* (Fig. 2g and Extended Data Fig. 2h).

Together, these data establish a conserved transcriptional zonation of mouse and human dermal microvasculature, revealing a pronounced post-capillary venous phenotype in venous dermal BECs.

### *Pik3ca*^*H1047R*^ drives venous proliferation and specification

**Fig. 3:**
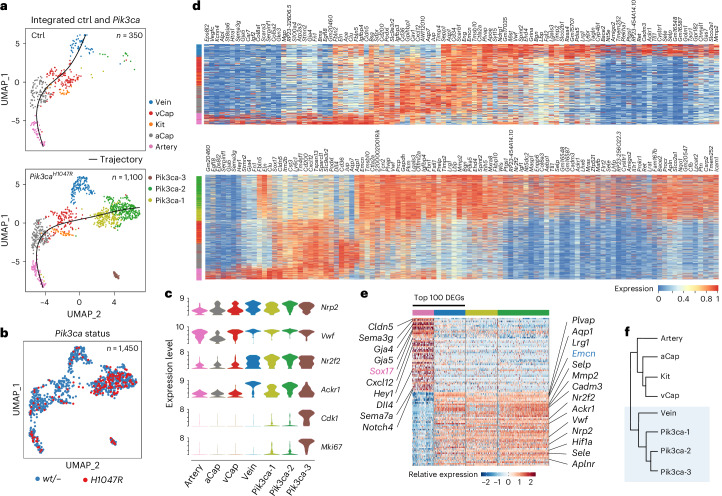
Venous identity of *Pik3ca*^*H1047R*^ -expressing BECs. **a**, Split UMAP representation of 1,450 single-cell transcriptomes of Harmony-integrated dermal BECs from Ctrl (350 cells, top) and *Pik3ca*^*H1047R*^*;Cdh5-CreER*^*T2*^ mice (1,100 cells, below) with color-coded cluster identities. Black lines show trajectories obtained by unsupervised trajectory interference analysis using Slingshot, visualized in UMAP space. **b**, *Pik3ca* transcript status visualized in combined UMAP clustering. **c**, Violin plots showing gene expression of selected venous and proliferation marker genes distributed across venous and disease-specific clusters. **d**, Heat maps of zonation marker expression across cells, ordered by cluster and along their trajectory, according to the respective UMAP representations for each genotype shown in **a**. Color indicates read counts on a log scale. **e**, Gene expression of the top 100 DEGs between artery and vein clusters in disease-specific clusters. Selected arterial (left) and venous (right) identity markers are highlighted. **f**, Cluster positions on a phylogenetic tree constructed in principal component analysis space using Seurat. The close relation of venous and disease-specific clusters is highlighted in blue. Source data

**Extended Data Fig. 3:**
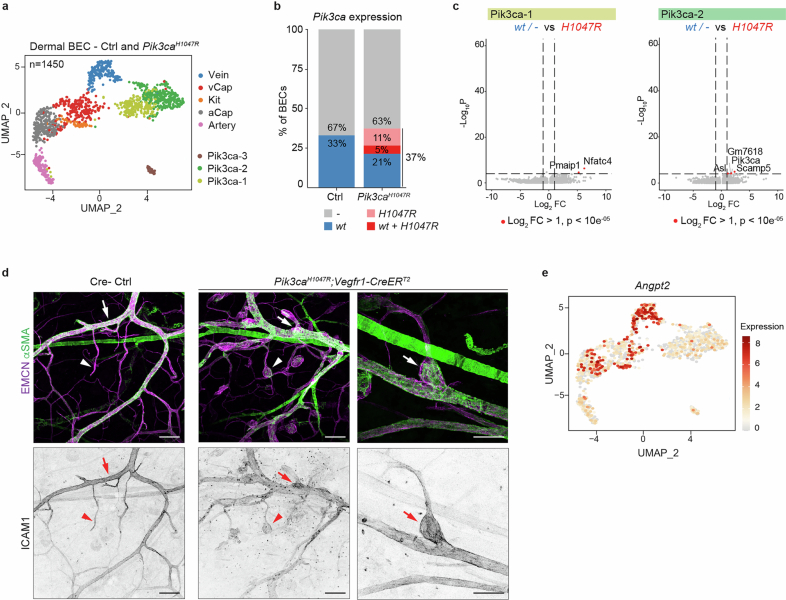
Characterization of *Pik3ca* mutant-specific BEC clusters. (**a**) Unsupervised trajectory inference analysis of dermal BECs from control and *Pik3ca* mutant mice using SLINGSHOT, visualized in a UMAP plot. (**b**) Proportion of BECs from control and *Pik3ca* mutant mice expressing either the endogenous mouse *Pik3ca* transcript (*wt*) and/or the transgenic *Pik3ca*^*H1047R*^ transcript (*H1047R*). (**c**) Volcano plots showing results from DEG analysis between *Pik3ca*^*H1047R*^ transcript-positive and -negative BECs from the mutant mice within Pik3ca-1 and Pik3ca-2 clusters. Negative log_2_ fold changes (FC) represent upregulated gene expression in *Pik3ca*^*H1047R*^ transcript-negative BECs (*wt* /-), while positive log_2_ FC represent upregulated gene expression in transcript-positive BECs. Significance of differential gene expression was assessed using Seurat’s Wilcoxon rank-sum test. Note only a few genes significantly upregulated in the transcript-positive BECs, marked in red (P-value < 0.00001, log_2_FC ≥ 1). (**d**) ICAM1 expression in EMCN^+^ venous ECs in whole-mount ear skin from 4-OHT-treated *Pik3ca*^*H1047R*^*;Vegfr1-CreER*^*T2*^ and Cre^-^ littermate control mice (Ctrl). αSMA is used to label SMCs. Arrows indicate larger veins; arrowheads point to venous capillaries. Similar results were obtained from 3 mice in 1 experiment. (**e**) UMAP representation of *Angpt2* transcript levels in BECs from control and *Pik3ca* mutant mouse. Scale bars: 50 μm. Source data

To identify *Pik3ca*^*H1047R*^-induced transcriptional changes in the dermal blood endothelium, we integrated BECs from the *Pik3ca* mutant and control mice. This integration revealed four additional clusters besides the artery, vein, aCap and vCap ECs observed in the control dataset (Fig. 3a and Extended Data Fig. 3a). One of these clusters included BECs from both genotypes and was characterized by the expression of *Kit* and markers of angiogenic tip cells (*Esm1*, *Apln*), as described for normal human and mouse BECs. In contrast, the remaining three clusters were almost exclusively composed of cells isolated from the mutant mice, thus termed Pik3ca-1–Pik3ca-3 (Fig. 3a) and enriched in cells expressing the mutant *Pik3ca*^*H1047R*^ transcript (Fig. 3b). In transgenic mice, mutant transcript-negative BECs either expressed the wild-type *Pik3ca* transcript or showed no transcript expression. However, as the wild-type *Pik3ca* transcript was detected in only approximately 30% of the BECs (Extended Data Fig. 3b), it is plausible that even the *Pik3ca*^*H1047R*^-negative BECs in the mutant-specific clusters are genetically mutant. Supporting this, comparisons between *Pik3ca*^*H1047R*^-negative and *Pik3ca*^*H1047R*^-positive BECs within Pik3ca-1 and Pik3ca-2 clusters showed minimal differences (Extended Data Fig. 3c). Pik3ca-3 represented proliferating BECs expressing cell cycle genes (for example, *Cdk1* and *Mki67*; Fig. 3c). Notably, the absence of BECs from the control mice in this cluster (Fig. 3a) indicates the quiescent state of normal mouse ear skin vasculature already at 5 weeks of age. Additional cluster markers and data are available at https://makinenlab.shinyapps.io/Mouse_DermalBloodEndothelialCells/.

Trajectory inference analysis of the integrated dataset revealed AV zonation in control BECs (Fig. 3a,d), in line with the data from control cells alone (Extended Data Fig. 2b). Interestingly, non-proliferative mutant ECs (Pik3ca-1 and Pik3ca-2) followed an alternative trajectory (Fig. 3a), and exhibited a venous gene signature including the expression of venous EC-identity markers (for example, *Nrp2*, *Nr2f2*, *Ackr1*; Fig. 3c,d). The venous phenotype of the mutant clusters was further substantiated through the analysis of the top 100 differentially expressed genes (DEGs) between control BECs within vein and artery clusters (Fig. 3e). In addition, unsupervised hierarchy tree construction demonstrated that the mutant BECs are closely related to BECs of venous identity (Fig. 3f).

### Clonal expansion of BECs with post-capillary venous phenotype

**Fig. 4:**
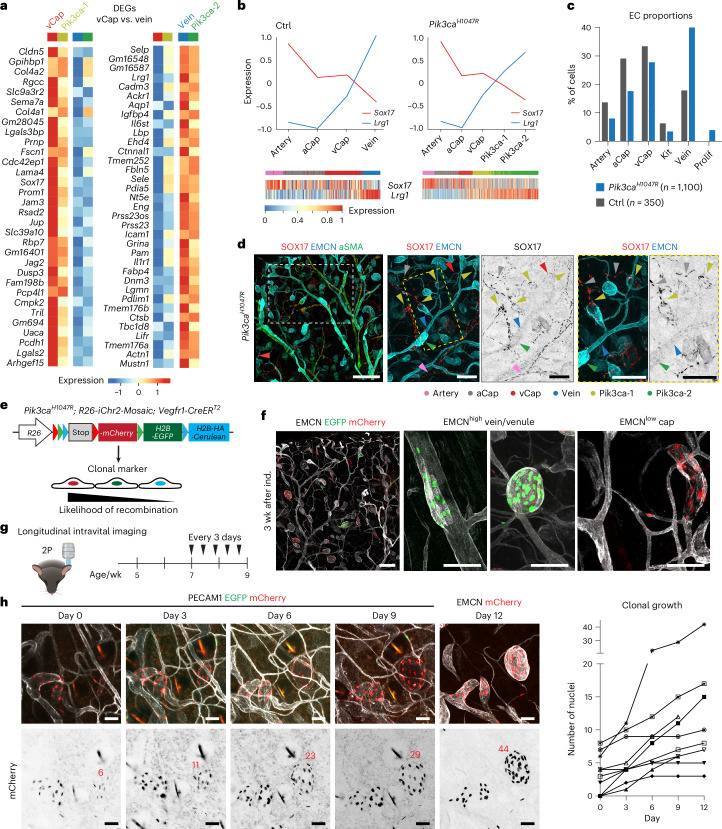
*Pik3ca*-driven VM is defined by clonal expansion of BECs with a post-capillary venous phenotype. **a**, Heat maps showing relative gene expression of selected top DEGs between vCap and vein clusters in non-proliferative disease-specific clusters. Color coding represents average relative expression across different clusters. **b**, Top, Average gene expression profile of exemplary arterial (*Sox17*, red) and venous (*Lrg1*, blue) markers in Ctrl or *Pik3ca* clusters. Below, Gene expression of *Sox17* and *Lrg1* in single cells, ordered along the Ctrl or *Pik3ca* trajectory shown in Fig. 3a. **c**, Bar graph of relative cell-type proportions split by genotype based on integrated BECs from *Pik3ca*^*H1047R*^*;Cdh5-CreER*^*T2*^ and Ctrl mice. **d**, Immunofluorescence of ear skin from a 4-OHT-treated 5-week-old *Pik3ca*^*H1047R*^*;Vegfr1-CreER*^*T2*^ mouse, showing vascular overgrowth in SOX17^+^EMCN^low^ capillaries (light-green arrowhead) and SOX17^−^EMCN^+^ veins (dark-green arrowhead). Phenotypically normal vessel types, annotated based on their morphology and marker expression, are highlighted by arrowheads as indicated. Similar results were obtained from two mice. **e**, Genetic construct for tamoxifen-inducible expression of a clonal *iChr2-Control-Mosaic* reporter in *Pik3ca*^*H1047R*^*;Vegfr1-CreER*^*T2*^ mice. Recombination occurs between arrowheads of the same color (LoxP site), resulting in the expression of one of three possible nuclear-localized fluorescent proteins. **f**, Whole-mount immunofluorescence of ear skin from 6-week-old *Pik3ca*^*H1047R*^*;R26-iChr2-Mosaic;Vegfr1-CreER*^*T2*^ mice with BECs expressing EGFP or mCherry. 4-OHT treatment (20 µg topically on the ear) was done at 3 weeks of age. Higher magnifications show representative EMCN^high^ veins of different calibers, and EMCN^low^ venous capillaries. Similar results were obtained from >5 mice in three independent experiments. **g**, Scheme for longitudinal intravital imaging of lesion growth in *Pik3ca*^*H1047R*^*;R26-iChr2-Mosaic;Vegfr1-CreER*^*T2*^ mice. **h**, Representative intravital microscopy images of the dermal microvasculature and BECs expressing EGFP or mCherry (on days 0, 3, 6 and 9), with nuclei counts indicated. PECAM1 antibody injection was used to visualize the vasculature. At the end of the experiment (day 12), the same lesions were imaged following whole-mount staining using confocal microscopy. **i**, Quantification of clonal expansion, showing the number of nuclei counted at five time points within the same lesions. Scale bars, 200 μm (**d**), 500 µm (**f**, overview), 100 μm (**f**, magnifications) and 50 μm (**h**, magnification). 2P, two-photon. Panel **e** adapted from ref. ^27^ under a Creative Commons license CC BY 4.0. Panel **g** created with BioRender.com. Source data

Next, we asked whether the transcriptional differences observed in BECs in the non-proliferative Pik3ca-1 and Pik3ca-2 clusters could be attributed to their distinct origins and anatomical locations within *Sox17*^*+*^*Emcn*^low^ capillaries and *Sox17*^−^*Emcn*^high^ veins. By focusing on genes that distinguish vCap from vein ECs, we found that Pik3ca-1 ECs share a transcriptional profile with vCap ECs, while Pik3ca-2 ECs are more similar to vein ECs. For example, Pik3ca-1 showed higher expression of markers enriched in vCap ECs, such as *Sox17*, compared to vein ECs (Fig. 4a). In contrast, (post-capillary) venous markers like *Selp*, *Lrg1*, *Ackr1* and *Icam1* were prominently expressed in the Pik3ca-2 cluster (Fig. 4a). The conserved zonation profile of the AV markers *Sox17* and *Lrg1* along the BEC trajectories between control and mutant mice (Fig. 4b) further indicated that the Pik3ca-1 and Pik3ca-2 clusters broadly displayed the transcriptional identities of vCap and vein ECs, respectively. The relative proportion of venous BECs was increased in *Pik3ca*^*H1047R*^ mutants at the expense of other BEC populations (Fig. 4c). Notably, the proportion of BECs expressing tip cell markers remained unchanged (Fig. 4c), in agreement with the lack of angiogenic sprouting in the mutants (Fig. 1b and ref. ^16^).

The identified expression profiles were also correlated with lesions located in different anatomical positions. Immunofluorescence staining of the ear skin from *Pik3ca*^*H1047R*^;*Vegfr1-CreER*^*T2*^ mice identified EMCN^high^ lesions as SOX17^−^ and ICAM1^+^ veins (Fig. 4d and Extended Data Fig. 3d), in line with their morphological and anatomical features, and thus matching the transcriptional profile of Pik3ca-2 BECs. In addition, we observed SOX17^+^EMCN^low^ lesions (Fig. 4d), as well as EMCN^low^ lesions expressing ICAM1 (Extended Data Fig. 3d), matching the transcriptional profile of Pik3ca-1. To analyze lesion growth dynamics, we tracked *Pik3ca*^*H1047R*^-expressing BECs using the *iChr2-Control-Mosaic* reporter^27^. This reporter allele allows multicolor clonal analysis, by generating fluorescently traceable clones expressing a single nuclear-localized fluorescent protein (mCherry, EGFP or mCerulean) upon Cre-mediated recombination (Fig. 4e). After the administration of 4-OHT to juvenile ear skin, recombination was observed in a subset of EMCN^high^ veins as well as EMCN^low^ capillaries (Fig. 4f). Most recombination events led to the expression of EGFP or mCherry, while Cerulean was observed rarely, in line with previous data^27^. The majority of lesions analyzed 3 weeks after 4-OHT induction (91% ± 2%, *n* = 192 lesions from four mice) showed just one fluorescent marker and were, therefore, likely monoclonal (Fig. 4f). Indeed, longitudinal intravital two-photon microscopy of the superficial capillary network confirmed clonal expansion as the primary mechanism driving lesion growth (Fig. 4g), with an average increase from 3 ± 3 cells at the start to 12 ± 11 by the end of the 12-day observation period (*n* = 8 lesions; Fig. 4h).

Collectively, these findings indicate that *Pik3ca*^*H1047*^ signaling drives clonal proliferation of BECs within capillaries and veins, and promotes the expansion of BEC populations with a post-capillary venous phenotype.

### Prevalent FOXO1-regulated transcription in *Pik3ca*^*H1047R*^ BECs

**Extended Data Fig. 4:**
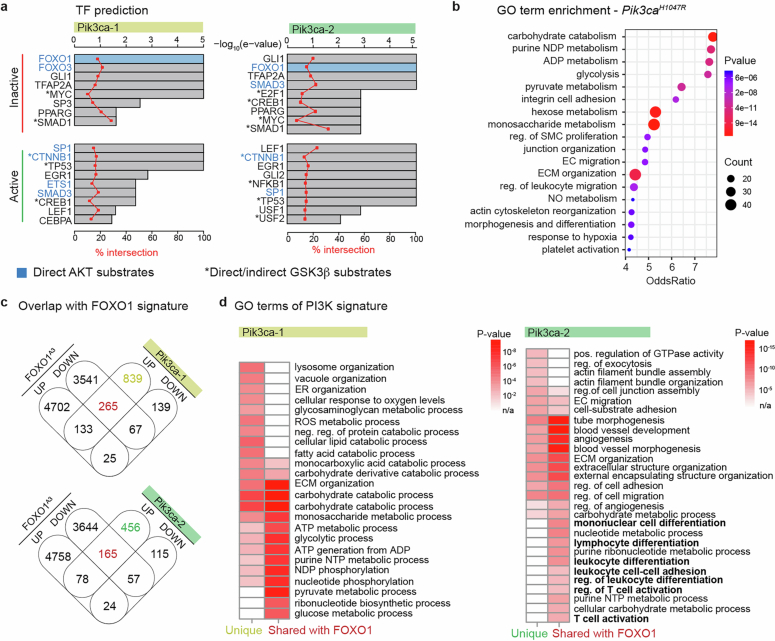
FOXO1-dependent and independent transcriptomes of *Pik3ca*^*H1047R*^. (**a**) Predicted transcription factors using TFactS based on up and down regulated DEGs (P < 0.00001, log2FC ≥ 1) of Pik3ca-1 and Pik3ca-2 compared to vCap and Vein clusters, respectively. Statistical significance is presented as e-value scores, red line represents intersection percentage between up- and downregulated genes for each transcription factor. Results were grouped by predicted activation or inactivation of transcription factors. Direct AKT substrates are indicated in blue, asterisks indicate GSK3β substrates. (**b**) GOstats enrichment analysis of top cluster markers (P < 0.00001, log2FC ≥ 1) of mutant-specific clusters Pik3ca-1 and Pik3ca-2. Top displayed GO terms are ordered by Odds Ratio, dot size represents gene count/GO term, P-values are encoded by colour gradient. (**c**) Venn diagrams showing shared and unique genes of DEGs significantly up- or downregulated (P < 0.00001) in HUVECs expressing AKT-resistant FOXO1^A3^ mutant compared to control HUVECs, and in mutant-specific BECs cluster Pik3ca-1 or Pik3ca-2 compared to vCap or Vein clusters. (**d**) GO enrichment analysis (GOstats) of upregulated DEGs unique for Pik3ca-1 or Pik3ca-2, and those shared with FOXO1 signature, as displayed in **c**. Terms associated with immune cell trafficking are indicated in bold. P-values of GO terms are encoded by colour gradient (n/a indicates no enrichment). Source data

Given the distinct gene expression changes in *Pik3ca*^*H1047*^ mutant ECs, we next attempted to predict transcription factors driving these cell-autonomous transcriptional programs. The prediction was based on gene expression differences between the mutant non-proliferative Pik3ca-1/Pik3ca-2 clusters and their wild-type counterparts. Transcription factors identified in both mutant clusters included substrates of PI3K–AKT signaling, such as FOXO1, SP1 and SMAD3 (Extended Data Fig. 4a). In addition, we identified transcription factors that are direct targets (for example, CTNNB1, MYC and TP53) or indirect targets (for example, LEF1) of the AKT-regulated glycogen synthase kinase-3 (GSK3B; Extended Data Fig. 4a), indicating that the observed transcriptional changes are closely linked to *Pik3ca*^*H1047*^ signaling. Consistent with this, GO term enrichment analysis showed metabolic regulation as a prominent shared biological response in the two mutant clusters, with biological processes involved in glycolysis and ADP metabolism among the top enriched terms (Extended Data Fig. 4b).

Given that FOXO1, the top hit in the transcription factor prediction, is inhibited by AKT through direct phosphorylation and serves as a central regulator of EC metabolism and growth^28^, we investigated its involvement in the *Pik3ca*-induced gene expression changes. To this end, we compared the PIK3CA-regulated genes to those regulated by a constitutively active, AKT-resistant FOXO1 mutant (FOXO1^A3^) in cultured human umbilical vein endothelial cells (HUVECs)^29^. Consistent with the opposing effects of PI3K and FOXO1, we found a substantial overlap between genes upregulated in *Pik3ca* mutant BECs with genes suppressed by FOXO1^A3^ (Extended Data Fig. 4c). GO term analysis of this PI3K-induced/FOXO1-suppressed gene signature showed enrichment of terms associated with metabolism and extracellular matrix organization in both Pik3ca clusters (Extended Data Fig. 4d). Additionally, immune-related terms were enriched specifically in the Pik3ca-2 cluster (Extended Data Fig. 4d). Conversely, terms enriched selectively in the *Pik3ca*-regulated but not in the FOXO^A3^ gene set included processes related to vesicular trafficking and actin organization (Extended Data Fig. 4d), suggesting that these known PI3K-regulated processes are FOXO1 independent. Together, these data are consistent with previous findings^28^ that FOXO1 is a crucial regulator of endothelial metabolism downstream of PI3K–AKT signaling. Furthermore, they suggest additional roles of PI3K–FOXO1 in governing the post-capillary venous phenotype in venous ECs.

### FOXO1-dependent ANGPT2 downregulation elevates TIE2 activity

**Fig. 5:**
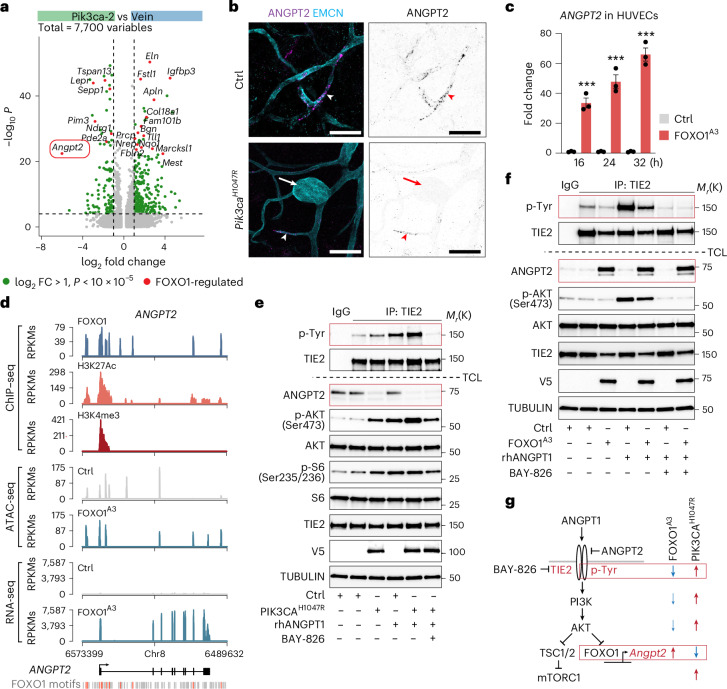
Loss of FOXO1-induced ANGPT2 expression and increase in TIE2 activity in *Pik3ca*^*H1047R*^*-*expressing BECs. **a**, Volcano plot of 7,700 DEGs between Pik3ca-2 and vein clusters. Negative log_2_ fold changes (*x* axis) represent downregulated gene expression, while positive log_2_ fold changes represent upregulated gene expression in the mutant. Differential gene expression was assessed using Seurat’s Wilcoxon rank-sum test. Significant upregulated and downregulated genes are marked in green (*P* value < 0.00001, log_2_FC ≥ 1; *y* axis) and those shared with FOXO1^A3^ downregulated genes (from **c**) are highlighted in red. **b**, Whole-mount immunofluorescence of mouse ear skin showing ANGPT2 in EMCN^+^ venous vessels (arrowheads), but not in vascular lesions in the *Pik3ca*^*H1047R*^ mutant mouse (arrow). Single-channel images for ANGPT2 staining are shown on the right. Similar results were obtained from three mice in two independent experiments. **c**, *ANGPT2* transcript levels in HUVECs transduced with AKT-resistant FOXO1^A3^ and Ctrl, analyzed by RNA-seq at different time points. Data points represent individual biological replicates of ANGPT2 mRNA expression levels (in fold change), mean ± s.d. (*n* = 3 (Ctrl) and *n* = 3 (FOXO1^A3^) at each time point). ***P < 0.001, unpaired two-tailed Student’s *t*-test: *P*(16 h) = 0.0006, *P*(24 h) = 0.0005, *P*(32 h) = 0.0001. **d**, ChIP–seq, ATAC-seq and RNA-seq signals at the *ANGPT2* genomic locus performed in FOXO1^A3^-expressing HUVECs. FOXO consensus motifs bound by FOXO1 are indicated in orange. Unbound FOXO motifs are shown in gray. Sequencing signals are represented as reads per kilobase million (RPKMs). **e**,**f**, Immunoblot analysis of immunoprecipitated TIE2 (top) or total cell lysates (TCL; below) from Ctrl HUVECs and HUVECs expressing *PIK3CA*^*H1047R*^ (**e**) or FOXO1^A3^ (**f**) using the indicated antibodies. AKT, S6, TIE2 and tubulin TCL western blots were used as sample loading controls. Cells were starved of serum and left untreated, or stimulated with ANGPT1 (50 ng ml^-1^ (**e**) or 200 ng ml^-1^ (**f**)), in the presence or absence of the TIE2 inhibitor BAY-826. *M*_r_(K) indicates protein molecular weight marker (in kDa). IgG isotype control was used as a negative control. Data are representative of two independent experiments. **g**, Illustration of the TIE2–PI3K–FOXO pathways (left) and effects of FOXO1 (via FOXO1^A3^ expression, middle) and PI3K activation (via *PIK3CA*^*H1047R*^ expression, right) on their pathway effectors. Blue indicates reduced activity; red indicates increased activity. Scale bars, 50 μm (**a**). Source data

We next focused on the Pik3ca-2 cluster that constituted the most expanded BEC population in the mutant vasculature (Fig. 4c). The top DEG between Pik3ca-2 and the corresponding normal vein ECs was *Angpt2*, which was predominantly expressed in venous BECs and downregulated in the mutant clusters (Fig. 5a and Extended Data Fig. 3e). In agreement with the transcriptomic data, immunofluorescence of the ear skin revealed ANGPT2 expression in EMCN^hi^ veins in control mice, as well as in phenotypically normal veins and tip ECs in the vascular sprouts of mutant *Pik3ca*^*H1047R*^ mice (Fig. 5b). In contrast, ANGPT2 expression was lost in vascular lesions in *Pik3ca*^*H1047R*^ mutant mice (Fig. 5b). Since ANGPT2 is regulated by FOXO1 (refs. ^30,31^), we assessed its expression in HUVECs expressing the PI3K–AKT-insensitive FOXO1^A3^ mutant. This analysis revealed a strong and time-dependent induction of *ANGPT2* transcript levels in FOXO1^A3^-expressing ECs (Fig. 5c). Moreover, studies of FOXO1 chromatin immunoprecipitation followed by sequencing (ChIP–seq) indicated that *ANGPT2* is a direct FOXO target gene, with FOXO1 binding to several canonical FOXO DNA-binding motifs in the *ANGPT2* genomic locus (Fig. 5d). Additionally, the activated state of the *ANGPT2* gene was indicated by acetylated histone H3 Lys27 (H3K27ac), trimethylated histone H3 Lys4 (H3K4me3) and an ‘open’ chromatin, revealed by the assay for transposase-accessible chromatin using sequencing (ATAC-seq; Fig. 5d), establishing *ANGPT2* as a bona fide FOXO1 target gene.

ANGPT2 is an autocrine context-dependent antagonist of TIE2 (ref. ^8^), an endothelial receptor tyrosine kinase frequently mutated in VMs^1^ and an upstream regulator of PI3K. To investigate the potential effect of PI3K-mediated *ANGPT2* regulation on TIE2 signaling, we analyzed TIE2 phosphorylation status in HUVECs expressing *PIK3CA*^*H1047R*^, with or without the agonistic ANGPT1 ligand. Immunoblot analysis with a phospho-tyrosine (pTyr)-specific antibody following TIE2 immunoprecipitation revealed low baseline phosphorylation of TIE2 in control HUVECs (Fig. 5e), potentially resulting from the expression of the TIE2 agonist ANGPT1 in these cells (bulkECexplorer^32^). This phosphorylation increased in HUVECs expressing *PIK3CA*^*H1047R*^ (Fig. 5e). Immunoblotting of total cell lysates confirmed downregulation of ANGPT2 and increased phosphorylation of the PI3K pathway targets AKT and ribosomal S6 in *PIK3CA*^*H1047R*^-expressing HUVECs (Fig. 5e). Mutant HUVECs also exhibited a stronger response to ANGPT1 stimulation compared to control HUVECs, with a greater increase in TIE2 and AKT phosphorylation, which was inhibited by treatment with the selective TIE2 inhibitor BAY-826 (Fig. 5e)^33^. In contrast, the expression of the constitutively active FOXO1^A3^ mutant resulted in high ANGPT2 levels and lower baseline TIE2 phosphorylation compared to control HUVECs (Fig. 5f). Additionally, FOXO1^A3^ expression reduced ANGPT1-induced phosphorylation of both TIE2 and AKT, which was also inhibited by BAY-826 (Fig. 5f).

Together, these findings indicate that TIE2 signaling, a major driver of VMs, is activated in *Pik3ca*^*H1047R*^-expressing venous ECs. Based on our in vitro data and the established role of PI3K–AKT signaling in FOXO inactivation, our results suggest that this activation is mediated, at least in part, by PI3K–AKT-induced FOXO1 inactivation and the resulting loss of the antagonistic ANGPT2 ligand (Fig. 5g).

### Elevated TIE2 phosphorylation in *Pik3ca*-driven VMs in mice

**Fig. 6:**
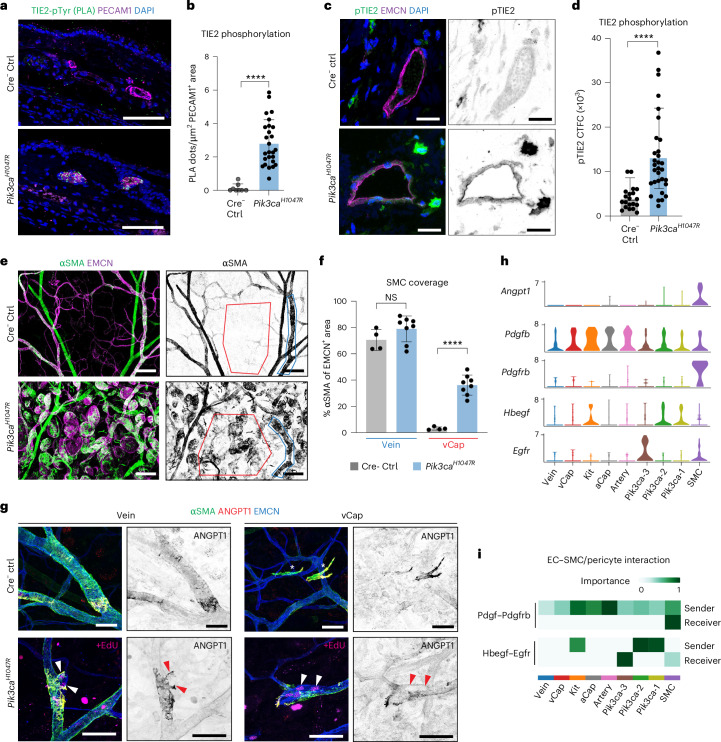
Increased TIE2 phosphorylation and SMC coverage in *Pik3ca*-driven VM in mice. **a**, PLA staining of activated TIE2 on ear skin paraffin sections from *Pik3ca*^*H1047R*^*;Vegfr1-CreER*^*T2*^ and Cre^−^ littermate Ctrl mice, detected using pTyr and TIE2 antibodies. DAPI marks cell nuclei. **b**, Quantification of PLA signals within PECAM1^+^ blood vessels. Data represent the number of PLA dots per μm^2^ of PECAM1^+^ vessel area, mean ± s.d. (*n* = 8 (Ctrl) and *n* = 26 (*Pik3ca*^*H1047R*^) vessels from four mice per genotype, unpaired two-tailed Student’s *t*-test, ****P = 0.0000054). **c**, Immunofluorescence of ear skin paraffin sections from *Pik3ca*^*H1047R*^*;Vegfr1-CreER*^*T2*^ and Cre^−^ littermate Ctrl mice using phospho-TIE2 antibodies. **d**, Phospho-TIE2 signal within EMCN^+^ vessels, represented as corrected total cell fluorescence (CTFC) of EMCN^+^ vessel area. Data points represent pTIE2 CTFC, mean ± s.d. (*n* = 20 (Ctrl) and *n* = 31 (*Pik3ca*^*H1047R*^) vessels from two mice per genotype, unpaired two-tailed Student’s *t*-test, ****P = 0.000096; Extended Data Fig. 8d). **e**, Whole-mount immunofluorescence of ear skin from *Pik3ca*^*H1047R*^*;Vegfr1-CreER*^*T2*^ and Cre^−^ littermate Ctrl mice using αSMA antibodies 6 weeks after 4-OHT induction. **f**, Quantification of SMC coverage of veins and capillaries, shown as average percentage of EMCN^+^ area, mean ± s.d. (*n* = 4 (Ctrl) and *n* = 8 (*Pik3ca*^*H1047R*^) mice, unpaired two-tailed Student’s *t*-test, *P*(vein) = 0.173 (NS, not significant) and ****P(capillary) = 0.0000078). **g**, Whole-mount immunofluorescence of ANGPT1^+^ cells associated with veins and capillaries one week after 4-OHT induction. Proliferating cells (arrowheads) were labeled in mutant mice with EdU 16 h before analysis. Asterisks indicate vessel-detached ANGPT1^+^ cells. **h**,**i**, Violin plots showing gene expression of *Angpt1* and selected marker genes of EC–SMC interaction (**h**) and heat map showing relative importance of two selected ligand–receptor pairs, generated using CellChat (**i**), in Ctrl and *Pik3ca* EC clusters from Fig. 3a as well as in SMCs from the same dataset. Scale bars, 50 μm (**a** and **g**), 20 μm (**c**) and 100 μm (**e**). Source data

**Extended Data Fig. 5:**
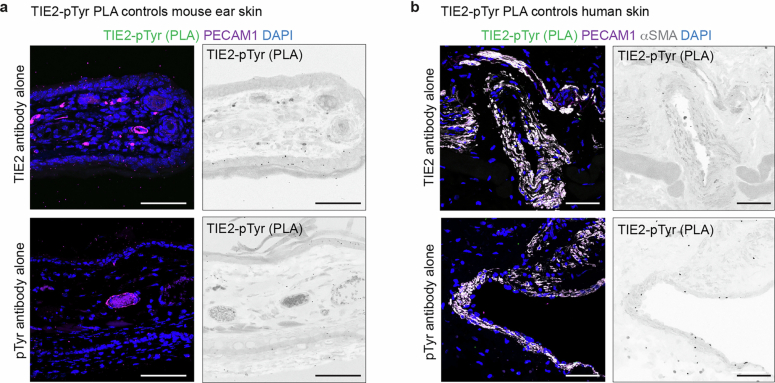
Technical controls for PLA. PLA analysis using either a phospho-Tyrosine (pTyr) or TIE2 antibody alone, showing the absence of signal (green) in mouse (**a**) and human (**b**) skin. PECAM1 and DAPI were used to mark blood endothelial cells and cell nuclei, respectively. αSMA marks smooth muscle cells. Scale bars: 50 μm (**a**, **b**).

Next, we assessed the activation status of TIE2 in the mutant vasculature using two complementary approaches. First, we performed in situ proximity ligation assay (PLA) on paraffin sections of mouse ear skin using antibodies against pTyr and the intracellular domain of TIE2. This analysis showed an increase in PLA signal in PECAM1^+^ vessels of the *Pik3ca*^*H1047R*^*;Vegfr1-CreER*^*T2*^ mutant mice in comparison to littermate controls (Fig. 6a,b and Extended Data Fig. 5a), indicating robust TIE2 activation. The low PLA signal in the control (Fig. 6a,b) suggests minimal baseline TIE2 activity in the quiescent dermal vasculature. To validate these findings, we performed immunofluorescence staining using a phospho-TIE2 antibody and quantified the signal intensity as a measure of phosphorylation level. Consistent with the PLA results, pTIE2 levels were markedly elevated in *Pik3ca*^*H1047R*^ mutant vessels compared to controls (Fig. 6c,d).

**Extended Data Fig. 6:**
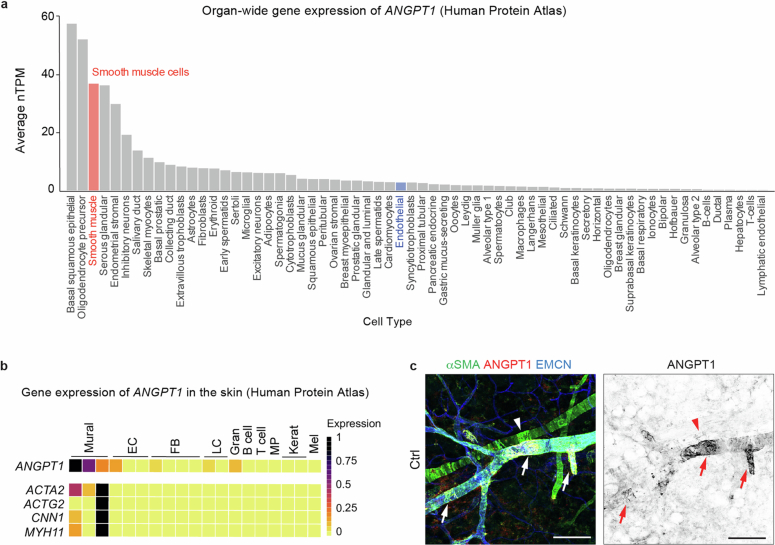
ANGPT1 expression in human and mouse tissues. (**a**) Organ-wide average gene expression (normalized transcripts per million, nTPM), of *ANGPT1* in different cell types, extracted from Human Protein Atlas scRNA-seq data. ANGPT1 expression in SMCs is highlighted in red and in BECs in blue. (**b**) Gene expression of *ANGPT1* and selected mural cell markers in different skin cell types extracted from Human Protein Atlas scRNA-seq data. (**c**) Whole mount immunofluorescence of ANGPT1^+^ SMCs (αSMA) associated with veins (arrows) but not arteries (arrowhead) in the ear skin of a control mouse. Similar results were obtained from 3 mice in 2 independent experiments. Scale bars: 50 μm.

Given that robust TIE2 activation requires agonist stimulation, we explored the Human Protein Atlas^34^ for potential cellular sources of ANGPT1. SMCs appeared as a primary source of ANGPT1 across multiple organs (Extended Data Fig. 6a) and specifically in the skin (Extended Data Fig. 6b). Importantly, vascular lesions in *Pik3ca* mutants exhibited prominent coverage by SMCs, with notable ectopic recruitment of SMCs to the dermal capillary bed (Fig. 6e,f and Extended Data Fig. 1c) as well as to the abnormal uterine vasculature (Extended Data Fig. 1c). Immunofluorescence labeling of control ear skin revealed a subset of SMCs expressing ANGPT1 specifically in veins, but not in arteries (Fig. 6g and Extended Data Fig. 6c). Additionally, alpha smooth muscle actin (αSMA)-positive cells were found detached from vessels, likely representing myofibroblasts (Fig. 6g). Interestingly, in the *Pik3ca* mutant vessels, ANGPT1-expressing SMCs surrounded actively growing lesions in both veins and venous capillaries, with proliferating ECs marked by EdU incorporation (Fig. 6g).

To investigate mechanisms that explain this aberrant SMC recruitment, we revisited the integrated scRNA-seq dataset of BECs from control and *Pik3ca* mutants, this time including the *Acta2*^*+*^*Pdgfrb*^*+*^ SMC cluster. Using CellChat, we interrogated interactions among all vascular cell types, including the SMC cluster (Fig. 6h). As expected from previous studies, the PDGF–PDGFRB interaction was identified as a general interaction between all ECs and SMCs, with the highest interaction scores observed between the *Kit*-EC and SMC clusters, as well as artery and SMC clusters (Fig. 6i). Moreover, HBEGF–EGFR interaction prediction was significant between the mutant *Pik3ca* clusters (Pik3ca-1 and Pik3ca-2) and SMCs, as well as between the *Kit*-EC and SMC clusters. Additionally, the proliferating Pik3ca-3 cluster was predicted as a significant HBEGF receiver population expressing *Egfr* (Fig. 6h,i).

Together, these findings show increased TIE2 phosphorylation in vivo in *Pik3ca*^*H1047R*^-expressing endothelium, and reveal ectopic recruitment of SMCs and their signaling interactions with ECs, including ANGPT1, as potential contributors to TIE2 activation and VM growth.

### Increased TIE2 phosphorylation in human VMs

**Fig. 7:**
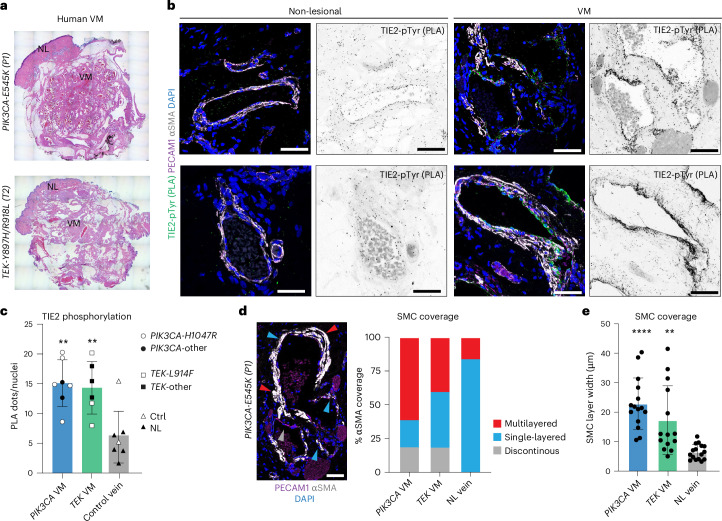
Increased TIE2 phosphorylation in human *PIK3CA*-driven VMs. **a**, H&E-stained paraffin sections of cutaneous VMs from individuals with *PIK3CA* (top) or *TEK* (below) mutations. Areas defined as non-lesional (NL) and VM lesions are indicated. **b**, PLA staining of activated TIE2, detected using pTyr and TIE2 antibodies, in representative vessels from indicated NL and VM regions. DAPI marks cell nuclei. **c**, Quantification of PLA signals within PECAM1^+^ veins, represented as mean PLA dots per EC nucleus ± s.d. (*n* = 7 (*PIK3CA*), *n* = 6 (*TEK*), *n* = 2 (Ctrl) and *n* = 5 (NL) individuals, with symbols indicating different mutations; ordinary one-way analysis of variance (ANOVA) and Tukey’s multiple-comparison test, **P(*PIK3CA* VM versus Ctrl vein) = 0.0027 and **P(*TEK* VM versus Ctrl vein) = 0.0078; Extended Data Fig. 7). **d**, Representative immunofluorescence image (left) and quantification (right) showing the proportion of SMC coverage in vessels from VM and NL regions, categorized into three groups: multilayered (>two rows of αSMA-associated nuclei; red), single-layered (1–2 rows of αSMA-associated nuclei; blue) and discontinuous (lack of SMC coverage; gray). Arrowheads indicate examples of each category. DAPI marks cell nuclei; αSMA marks SMCs. **e**, Quantification of the SMC layer thickness in *PIK3CA* and *TEK* individuals, presented as the mean ± s.d. (*PIK3CA* individuals P1 (*n* = 11) and P2 (*n* = 4 vessels); *TEK* individuals T1 (*n* = 3), T2 (*n* = 3) and T3 (*n* = 8 vessels); NL regions P1 (*n* = 4), P2 (*n* = 4), T1 (*n* = 5) and T3 (*n* = 3 vessels)). Ordinary one-way ANOVA and Tukey’s multiple-comparison test, ****P(*PIK3CA* VM versus NL vein) = 0.0000071 and **P(*TEK* VM versus NL vein) = 0.003. Scale bars, 50 μm (**b** and **d**). Source data

**Extended Data Fig. 7:**
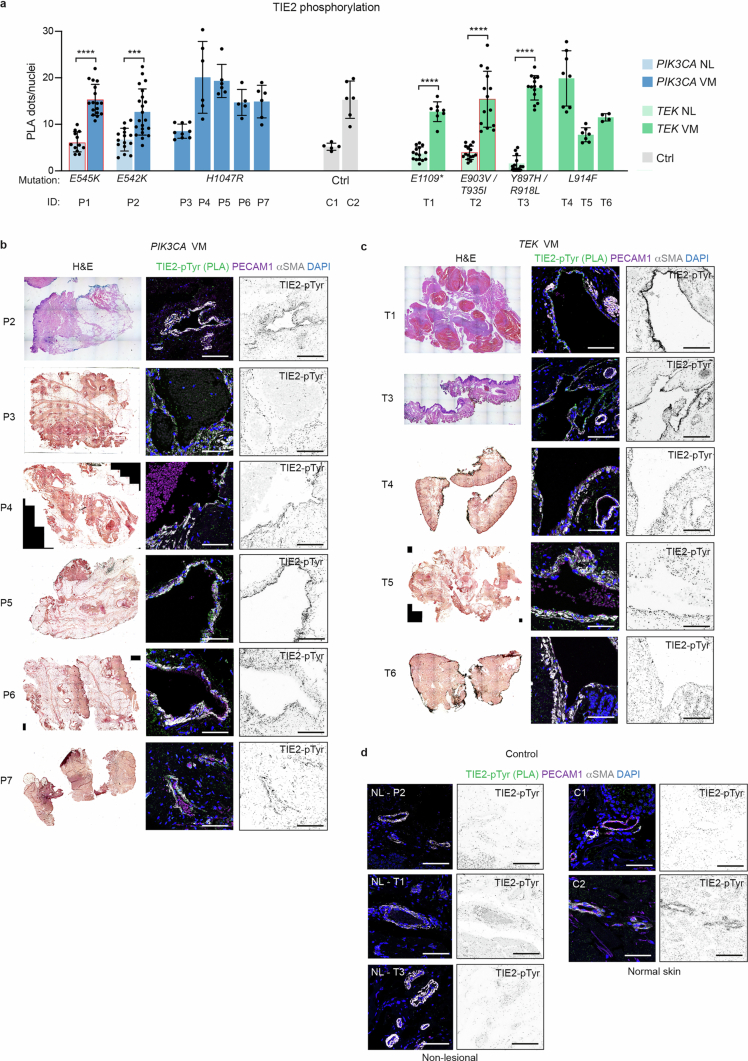
TIE2 phosphorylation in human VM. (**a**) Quantification of PLA signals of activated TIE2, detected using pTyr and TIE2 antibodies, within PECAM1^+^ veins from individual patients and healthy individuals (Ctrl) from two different cohorts, denoted by patient ID (see Supplementary Table 1 for details). Data represent number of PLA dots per nucleus of individual vessels, mean ± sd (*PIK3CA* patients P1 (n = 17), P2 (n = 21), P3 (n = 9), P4 (n = 6), P5 (n = 6), P6 (n = 5), P7 (n = 6) vessels; *TEK* patients T1 (n = 8), T2 (n = 14), and T3 (n = 14), T4 (n = 8), T5 (n = 7), T6 (n = 4) vessels; Healthy individuals C1 (n = 5), C2 (n = 6) vessels; NL regions P1 (n = 12), P2 (n = 4), T1 (n = 15), and T3 (n = 15) vessels). Barplots outlined in red indicate patients shown in Fig. 7a, b. ***P < 0.001, ****P < 0.0001, unpaired two-tailed Student’s t-test; Exact P values are provided in Source Data. (**b-d**) Hematoxylin and Eosin (H&E)-stained paraffin sections of cutaneous VMs from patients with *PIK3CA* (**b**) or *TEK* mutations (**c**), or of control skin (**d**) including non-lesional (NL) area and normal skin as indicated. PLA staining of activated TIE2, detected using pTyr and TIE2 antibodies, in representative vessels are shown on the right. DAPI marks cell nuclei. Scale bars: 50 μm (**b-d**). Source data

To assess the clinical relevance of the findings, we analyzed TIE2 phosphorylation in biopsy samples of skin from 13 individuals with VMs. The clinical features of the selected individuals with activating *PIK3CA* mutations and those with *TEK* mutations, used as positive controls, are summarized in Supplementary Table 1. In addition, skin samples from two healthy individuals were included for comparison. Paraffin-embedded skin sections were stained with hematoxylin and eosin (H&E) to differentiate between normal non-lesional (NL) tissue and lesion regions (VM; Fig. 7a), and PLA was performed on adjacent sections to compare these regions. As expected, PECAM1^+^ lesional vessels in most individuals with activating *TEK* mutations (4 of 6) showed a strong increase in TIE2-pTyr PLA signals compared to the adjacent NL tissue and tissue from healthy individuals (Fig. 7b,c and Extended Data Fig. 7a–d and Extended Data Fig. 5b). A similar increase in TIE2 phosphorylation was observed in individuals with *PIK3CA* mutations, including the p.His1047Arg (4 of 5) and p.Glu542Lys or p.Glu545Lys (2 of 2) mutations (Fig. 7b,c and Extended Data Fig. 7a–d). Additionally, we observed prominent, although irregular, SMC coverage around lesions in both groups (Fig. 7d,e). In both *PIK3CA* and *TEK* VMs, approximately 20% of the vessel area lacked SMC coverage (Fig. 7d). However, in *PIK3CA* mutant VMs, around 60% of the vessel area was covered by multiple layers of SMCs, compared to 40% in *TEK*-related VMs (Fig. 7d), which was accompanied by a significant increase in the overall width of the SMC layer (Fig. 7e). These results suggest that increased TIE2 activity and uncontrolled SMC recruitment may contribute to the pathogenesis of human VMs caused by *PIK3CA* mutations.

### TIE2 inhibition limits *Pik3ca*-driven VM growth

**Extended Data Fig. 8:**
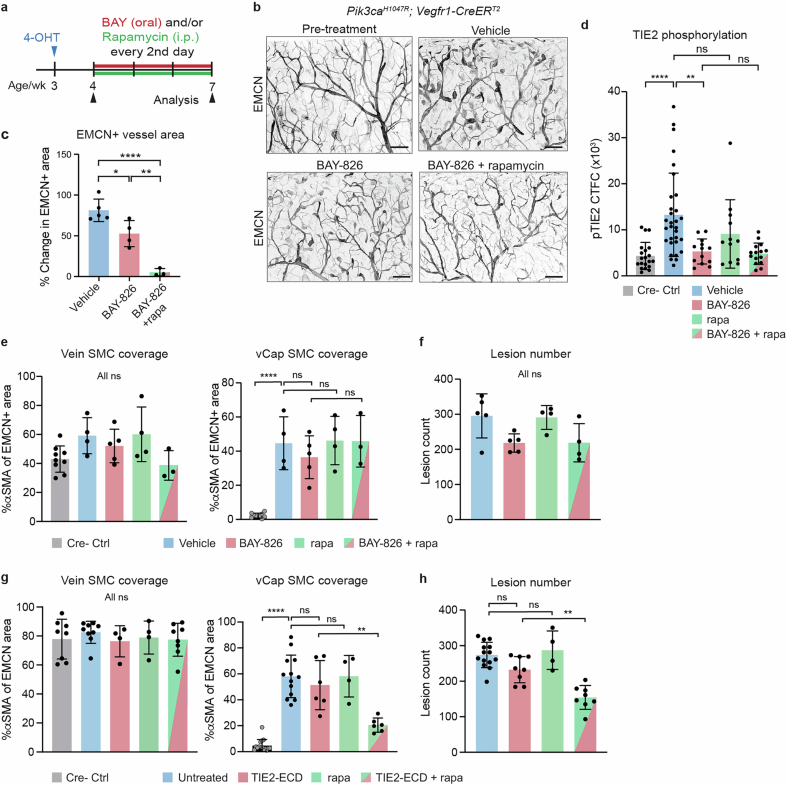
Effects of BAY-826/rapamycin on *Pik3ca*-driven VM lesions. (**a**) Experimental scheme for preventive treatment of *Pik3ca*-driven VM with BAY-826 (50 mg/kg by oral gavage) and in combination with rapamycin (rapa, 10 mg/kg by intraperitoneal injection, i.p.). (**b**) Whole-mount immunofluorescence of ear skin from *Pik3ca*^*H1047R*^*;Vegfr1-CreER*^*T2*^ mice at treatment start (one week after 4-OHT induction), and after a three-week treatment period. (**c**) Treatment effect on vessel growth, shown as increase in EMCN^+^ vessel area relative to Cre^-^ littermate control, mean ± sd (n = 5 (Vehicle), n = 4 (BAY-826), n = 3 (BAY-826+rapa) mice), ordinary one-way ANOVA and Tukey’s multiple comparison P(Vehicle vs. BAY-826) = 0.000065 and P(Vehicle vs. BAY-826 + rapa)= 0.0029. (**d**) Phospho-TIE2 signal within EMCN^+^ vessels in *Pik3ca*^*H1047R*^*;Vegfr1-CreER*^*T2*^ and Cre^-^ littermate control mice (Ctrl) after BAY-826 and/or rapamycin treatment, following the scheme in Fig. 8a, represented as corrected total cell fluorescence (CTFC) of EMCN^+^ vessel area, mean ± sd (n = 20 (Ctrl); Cre^+^ cohorts: n = 31 (Vehicle), n = 31 (BAY-826), n = 12 (rapa), n = 15 (BAY-826+rapa) vessels from 2 mice per group). (**e**) SMC coverage in ear skin of *Pik3ca*^*H1047R*^*;Vegfr1-CreER*^*T2*^ and Cre^-^ littermate control mice, shown as average % of EMCN^+^ area ± sd of veins and capillaries (n = 9 (Ctrl); Cre^+^ cohorts: n = 4 (Vehicle), n = 5 (BAY-826), n = 4 (rapa), n = 3 (BAY-826+rapa) mice). (**f**) Lesion numbers in the ear skin of *Pik3ca*^*H1047R*^*;Vegfr1-CreER*^*T2*^ mice, mean ± sd (n = 5 (Vehicle), n = 5 (BAY-826), n = 4 (rapa), n = 4 (BAY-826+rapa) mice). (**g**) SMC coverage in the ear skin of *Pik3ca*^*H1047R*^*;Vegfr1-CreER*^*T2*^ and Cre^-^ littermate control mice (Ctrl), shown as average % of EMCN^+^ area ± sd of veins (n = 8 (Ctrl); Cre^+^ cohorts: n = 9 (untreated), n = 4 (TIE2-ECD), n = 4 (rapa), n = 7 (TIE2-ECD+rapa) mice) and capillaries (n = 15 (Ctrl); Cre^+^ cohorts: n = 13 (untreated), n = 6 (TIE2-ECD), n = 4 (rapa), n = 7 (TIE2-ECD+rapa) mice). (**h**) Lesion numbers in the ear skin of *Pik3ca*^*H1047R*^*;Vegfr1-CreER*^*T2*^ mice, mean ± sd (n = 14 (untreated), n = 8 (TIE2-ECD), n = 4 (rapa), n = 7 (TIE2-ECD+rapa) mice); Differences in absolute levels of % SMC coverage in **e** and **g** is due to differences in image acquisition parameters. *P < 0.05, **P < 0.01, ****P < 0.0001, ns, P > 0.05, Ordinary one-way ANOVA and Tukey’s multiple comparison. Exact P values are provided in Source Data. Scale bars: 500 μm (**b**). Source data

**Extended Data Fig. 9:**
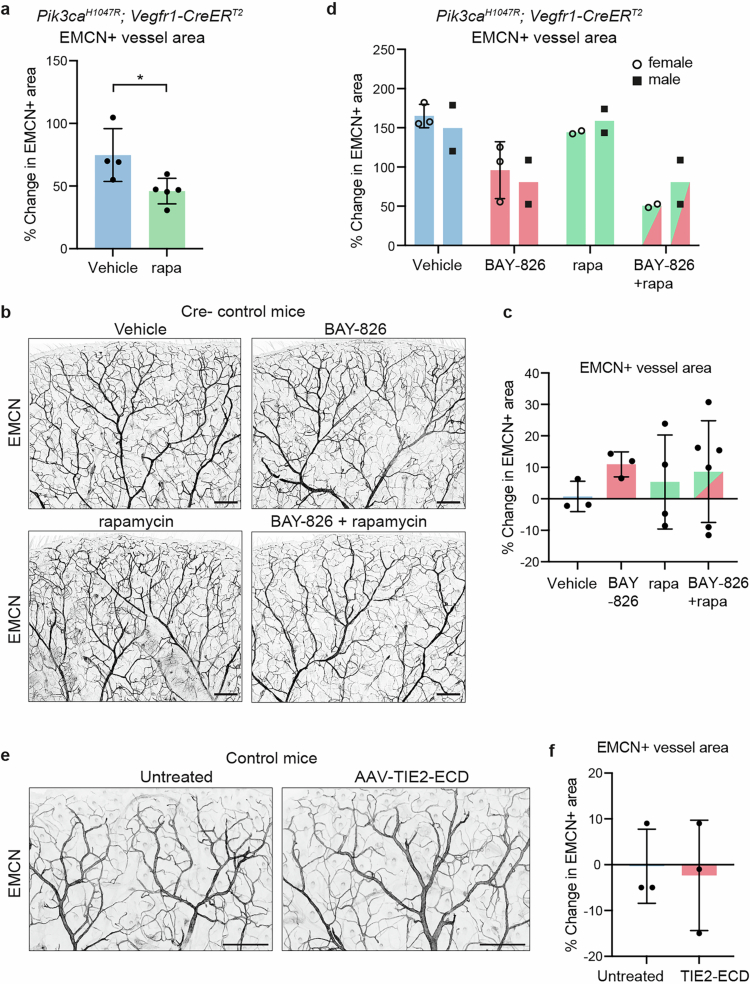
Effects of treatment on early lesions and control mice. (**a**) Outcome of rapamycin treatment during early lesion formation in *Pik3ca*^*H1047R*^;*Vegfr1-CreER*^*T2*^ mice (4-OHT induction at 3 weeks, treatment start at 4 weeks for 5 consecutive days, analysis at 5 weeks). Data represent increase in EMCN^+^ vessel area relative to Cre- littermate control, mean ± sd (n = 4 (Vehicle), n = 5 (rapa) mice), P = 0.0302 (*), unpaired two-tailed Student’s t-test. (**b, c**) Representative whole-mount immunofluorescence images of dermal vasculature of ear skin (**b**) and quantification (**c**) of the effect of TIE2 inhibitor BAY-826 and/or rapamycin treatment on Cre^-^ littermate control mice. Treatment scheme is as in Fig. 8a (4-OHT induction at 3 weeks, treatment start at 6 weeks, analysis at 8 weeks). Data in (**c**) represent increase in EMCN^+^ vessel area relative to Vehicle control, mean ± sd (n = 3 (Vehicle), n = 4 (rapa), n = 3 (BAY-826), n = 6 (BAY-826+rapa) mice). (**d**) Treatment outcome in *Pik3ca*^*H1047R*^;*Vegfr1-CreER*^*T2*^ mice by sex. Data shown in Fig. 8c is split by sex and represent increase in EMCN^+^ vessel area relative to Cre^-^ littermate control mice of the same sex, mean ± sd (n = 5 (Vehicle), n = 5 (BAY-826), n = 4 (rapa), n = 4 (BAY-826+rapa) mice). (**e**, **f**) Representative whole-mount immunofluorescence images of dermal vasculature of ear skin (**e**) and quantification (**f**) of the effect of AAV-TIE2-ECD on control mice not carrying Cre or *Pik3ca*^*H1047*^ alleles. Treatment scheme is as described in Fig. 8e (AAV at 3 weeks, 4-OHT induction at 4 weeks, analysis at 8 weeks). Data in (**f**) represent a change in EMCN^+^ vessel area relative to untreated control, mean ± sd (n = 3 (untreated), n = 3 (TIE2-ECD) mice). Scale bars: 200 μm (**b, e**). Source data

To explore the therapeutic potential of TIE2 inhibition in VMs, we treated *Pik3ca*^*H1047R*^*;Vegfr1-CreER*^*T2*^ mice with the selective TIE2 inhibitor BAY-826 alone or in combination with the clinically used mTOR inhibitor rapamycin. First, we initiated TIE2 inhibition at the onset of lesion induction, one week after 4-OHT application, and analyzed ear skin after 3 weeks of treatment with BAY-826 (Extended Data Fig. 8a,b). Whole-mount immunofluorescence revealed modest inhibition of EMCN^+^ vessel growth in mice treated with BAY-826 compared to vehicle-treated controls (Extended Data Fig. 8b,c). As previously demonstrated^35^, intraperitoneal administration of the mTOR inhibitor rapamycin also inhibited VM growth (Extended Data Fig. 9a). Notably, combined administration of BAY-826 and rapamycin prevented lesion formation (Extended Data Fig. 8b,c), while treated Cre^−^ control littermates showed no apparent vascular alterations (Extended Data Fig. 9b,c), and no significant differences between the sexes were observed (Extended Data Fig. 9d).

**Fig. 8:**
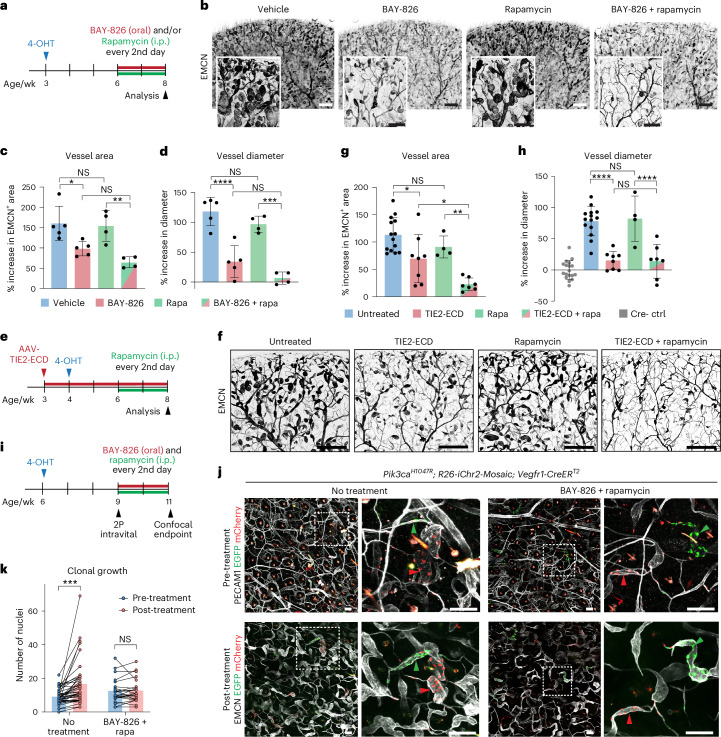
Inhibition of the upstream TIE2 receptor signaling limits *Pik3ca*^*H1047R*^ -driven VM growth. **a**, Experimental scheme for therapeutic treatment of advanced *Pik3ca*-driven VM with the TIE2 inhibitor BAY-826 (50 mg per kg body weight by oral gavage) and/or rapamycin (rapa, 10 mg per kg body weight by intraperitoneal (i.p.) injection). **b**, Whole-mount immunofluorescence of ear skin from *Pik3ca*^*H1047R*^*;Vegfr1-CreER*^*T2*^ mice with advanced VMs after a 2-week treatment period. **c**,**d**, Quantification of the treatment outcome. Bar plots show the increase in EMCN^+^ vessel area relative to Cre^−^ littermate Ctrl mice, mean ± s.d. (*n* = 5 (vehicle), *n* = 5 (BAY-826), *n* = 4 (rapa), *n* = 7 (BAY-826 + rapa) mice; **c**); or increase in vessel diameter relative to Cre^−^ littermate controls, mean ± s.d. (*n* = 5 (vehicle), *n* = 5 (BAY-826), *n* = 4 (rapa), *n* = 4 (BAY-826 + rapa) mice (**d**). **e**, Experimental scheme for the induction of VMs and inhibition of TIE2 signaling by intraperitoneal injection of AAV vectors encoding a ligand-neutralizing soluble TIE2 extracellular domain (TIE2-ECD). **f**–**h**, Whole-mount immunofluorescence of ear skin from *Pik3ca*^*H1047R*^*;Vegfr1-CreER*^*T2*^ mice (**f**) and quantification of the treatment outcome (**g** and **h**). Bar plots show the increase in EMCN^+^ vessel area relative to Cre^−^ littermate Ctrl mice, mean ± s.d. (*n* = 14 (untreated), *n* = 8 (TIE2-ECD), *n* = 4 (rapa), *n* = 7 (TIE2-ECD + rapa) mice; **g**); or increase in vessel diameter relative to Cre^−^ littermate Ctrl mice, mean ± s.d. (*n* = 18 (Ctrl); Cre^+^ cohorts: *n* = 14 (untreated), *n* = 8 (TIE2-ECD), *n* = 4 (rapa), *n* = 7 (TIE2-ECD + rapa) mice (**h**). **i**, Scheme for assessing BAY-826 treatment response in *Pik3ca*^*H1047R*^*;R26-iChr2-Mosaic;Vegfr1-CreER*^*T2*^ mice. **j**, Top, Intravital two-photon (2P) microscopy images of the dermal microvasculature stained using intravenous PECAM1 antibody injection, showing clonal lesions expressing EGFP or mCherry, at the start of treatment period. Below, Confocal images of the same lesions after a 2-week treatment period. Boxed regions are magnified on the right. **k**, Quantification of clonal expansion showing pretreatment and post-treatment nuclei counts (*n* = 42 lesions from six Ctrl mice and *n* = 24 lesions from three BAY-836-treated mice). In **c**, **d**, **g**, **h** and **k**, *P < 0.05, **P < 0.01, ****P < 0.0001, ordinary one-way ANOVA and Tukey’s multiple-comparison test (**c**, **d**, **g** and **h**) and paired two-tailed Student’s *t*-test (**k**). Scale bars, 100 μm (**b** and **j**, magnification) and 500 μm (**b**, **f** and **j**, overview). Source data

To test the therapeutic effect of BAY-826 on advanced VM lesions, the treatment was initiated at 3 weeks after 4-OHT induction and administered every other day (Fig. 8a). Analyzing the ear skin following a 2-week treatment regimen showed a minimal effect of rapamycin on vascular growth when compared to the respective vehicle control (Fig. 8b,c). In contrast, oral BAY-826 reduced the EMCN^+^ vascular area by 39% compared to the vehicle control (Fig. 8c). This reduction increased to 60% when TIE2 inhibition was combined with rapamycin (Fig. 8c). Immunostaining confirmed reduced TIE2 phosphorylation in BAY-826-treated and combination-treated mice (Extended Data Fig. 8d). While BAY-826 treatment did not significantly affect SMC coverage of veins or recruitment to the capillary bed (Extended Data Fig. 8e) or lesion numbers (Extended Data Fig. 8f), it significantly decreased venous vessel diameter—by 71% alone and by 94% in combination with rapamycin—a phenotypic outcome not observed with rapamycin alone (Fig. 8d).

To further substantiate these data, we used AAV vectors encoding a soluble TIE2 extracellular domain (AAV9-mTIE2-ECD)^36^ to neutralize the ANGPT ligands (Fig. 8e). AAVs were administered one week before 4-OHT induction, and rapamycin treatment started 2 weeks after induction for a 2-week period (Fig. 8e). Ligand neutralization alone significantly reduced vascular area in most mice, with the greatest effect achieved when combined with rapamycin (Fig. 8f,g), while the decrease in vessel diameter was similar between the two treatments (Fig. 8h). Notably, the combined ligand-neutralizing and rapamycin treatment also led to a significant decrease in SMC coverage of mutant capillaries and lesion numbers, while veins remained unaffected (Extended Data Fig. 8g). Control mice treated with AAV-TIE2-ECD showed no apparent vascular alterations (Extended Data Fig. 9e,f).

Next, we performed intravital two-photon imaging of lesions before and after BAY-826/rapamycin treatment to study the dynamics of lesion growth. While most lesions continued to grow in untreated mice, they stopped growing or even decreased in cell number in those administered with the combinatorial treatment (Fig. 8j,k).

Collectively, these findings demonstrate that inhibition of upstream TIE2 signaling effectively suppresses *Pik3ca*-driven VM growth, in particular when combined with rapamycin, suggesting a promising therapeutic strategy for treating advanced VMs.

## Discussion

**Extended Data Fig. 10:**
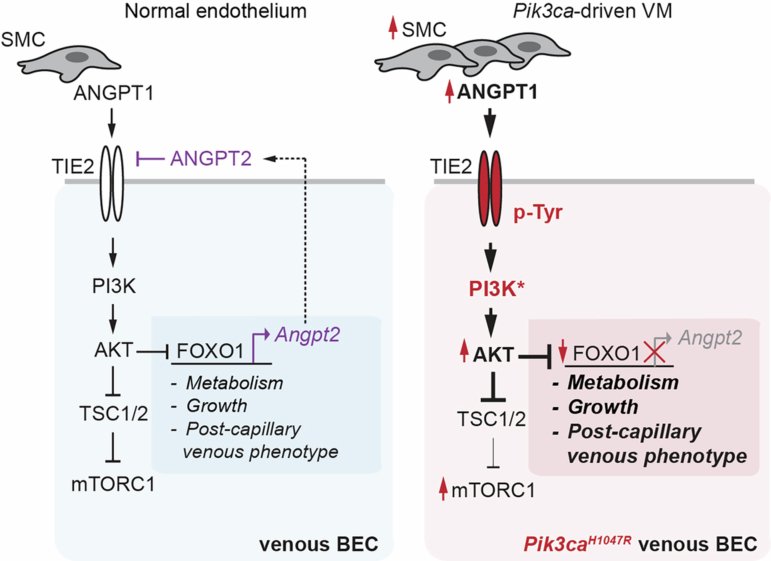
TIE2-PI3K-FOXO1 signaling pathway and its dysregulation in *Pik3ca*-driven VM. PI3K-AKT pathway regulates diverse cellular processes including protein translation (via TSC1/2-mTORC1) and gene transcription through direct (for example, inhibition of FOXO1) or indirect (for example, activation of β-catenin through inhibition of GSK3β) mechanisms. Examples of other AKT targets involved for example, in the regulation of cell survival (BAD) and vascular tone (eNOS) are indicated. FOXO1 inhibition-dependent PI3K signaling contributes to regulation of cellular metabolism, vascular growth and the acquisition of post-capillary venous phenotype. Activation of the PI3K pathway results in “feedforward” signaling to TIE2 by suppressing FOXO1-regulated expression of the autocrine TIE2 antagonist, ANGPT2. The resulting upstream activation of PI3K signaling is reinforced by an increase in coverage by smooth muscle cells (SMCs) that produce the TIE2 agonist, ANGPT1.

VMs frequently arise from mutations that lead to aberrant activation of the endothelial TIE2 receptor, a critical regulator of normal venous differentiation and growth. VMs can also result from activating mutations in the ubiquitous downstream effector PI3Kα, which is normally activated by TIE2 and various growth factor receptors. In this study, we uncovered a venous-specific signaling circuit involving a PI3K–FOXO1–ANGPT–TIE2 cascade that contributes to the vessel-type selective development of PI3Kα-driven vascular malformations. In mutant venous BECs expressing constitutively active *Pik3ca*^*H1047R*^, reduced ANGPT2 expression results from AKT-mediated inactivation of its transcriptional regulator, FOXO1 (Extended Data Fig. 10). This coincided with ectopic recruitment of SMCs expressing the TIE2 agonist ANGPT1 and increased TIE2 activity. Notably, TIE2 inhibition or ligand neutralization was more efficient than rapamycin in suppressing the growth of advanced VM lesions, suggesting a potential therapeutic approach for treating *PIK3CA*-related venous overgrowth.

VMs are highly prevalent in the skin and subcutaneous tissue^1^. Susceptibility of the skin to *Pik3ca*-driven VM formation was recapitulated in mice upon BEC-specific expression of the disease-causative *Pik3ca*^*H1047*^ mutation, highlighting the clinical relevance of the model used in this study. In line with previous findings^16^, we found that the development of cutaneous VM lesions occurred in the absence of developmental growth, and could even be induced in mature quiescent vasculature. Interestingly, we found that VM lesion development was not inhibited by the blockade of the major angiogenic regulator VEGF, suggesting involvement of other growth factors. This is in contrast to cutaneous lymphatic malformations, where the related lymphangiogenic growth factor VEGFC is essential for *Pik3ca*-driven vessel overgrowth^16^.

Using single-cell transcriptomics, zonation along the AV axis within the dermal microvasculature in both mouse and human data was identified. Conservation of markers in the mouse vasculature, including typical post-capillary venule markers (for example, *ICAM1* and *SELE*) and pre-venular capillary markers (SOX17), was demonstrated, consistent with findings previously reported in human skin^26^. Notably, all venous ECs in our dataset, making up 18% of the total dermal EC population, exhibited a molecular signature typical of post-capillary venules, which are the major sites for leukocyte extravasation. In contrast, the brain vasculature has a similar proportion of venous ECs, but only 3% of the EC population exhibits a post-capillary venous phenotype^37^. This underscores the presence of tissue-specific molecular and functional traits related to immune cell trafficking that may contribute to the unique responsiveness of dermal veins to *Pik3ca*-driven overgrowth.

Analysis of single-cell transcriptomes and clonal tracing of BECs showed a selective expansion of venous and venous capillary ECs in the *Pik3ca* mutant mice. While the differences in disease pathology between the two lesion types remain incompletely understood, it is plausible that, like normal veins and venous capillaries, they have different abilities to regulate immune responses and control vascular leakage depending on their vessel of origin. Such differences could have critical implications for disease progression and therapeutic responses. Predicted transcription factor activity, based on *Pik3ca*-driven gene expression changes within these EC populations, identified the AKT-inhibited transcription factor FOXO1 as a central regulator of the PI3K-driven metabolic program and post-capillary venous traits. Intriguingly, a prominent expression of FOXO1 was shown previously in human skin post-capillary venules^26^. Of particular interest, we showed that the antagonistic TIE2 ligand, ANGPT2, is a bona fide direct FOXO1 target gene, which is transcriptionally induced directly upon FOXO1 activation. ANGPT2 was expressed in dermal veins but absent in *Pik3ca*-driven VM lesions. Similarly, inhibition of FOXO1 and *ANGPT2* transcription has been reported in HUVECs expressing the TIE2 overactivating mutation p.Leu914Phe^38^. *Pik3ca*-driven VM lesions showed an increased presence of SMCs producing the TIE2 agonist ANGPT1 (ref. ^39^ and this study), along with an increase in venous EC TIE2 activation that was also detected in biopsy samples of cutaneous *PIK3CA*-related human VMs. These results suggest a shift in the balance of TIE2-activating ligands, a key regulatory mechanism for venous differentiation and growth^7^, which can also promote the transformation of inflamed capillaries into venules primed for leukocyte trafficking^40^. The post-capillary venous characteristics of the *Pik3ca*-driven lesions may, in turn, promote leukocyte influx and inflammation, which has increasingly been recognized as an important component of the pathogenesis of different types of vascular malformations^41,42^. In support of these notions, our previous analyses of a mouse model of advanced *Pik3ca*-driven cutaneous VMs revealed infiltration of B cells and neutrophils^16^, which can produce ANGPT1 (ref. ^40^) and thereby further amplify TIE2 signaling.

Rapamycin, which targets the AKT downstream effector mTORC1, has shown success in the clinical treatment of VMs, although its effectiveness can vary^1^. In particular, treatment duration and timing may impact outcomes. Most clinical studies involve long-term rapamycin treatment to manage established lesions, whereas better overall efficacy is observed when therapy is initiated early^43,44^. This improved effect is likely due to its antiproliferative action on actively growing lesions, inhibiting cellular anabolism, protein synthesis and growth. The short-term rapamycin treatment scheme used in our study, designed for a rapidly developing mouse model of VM, differs from clinical treatment regimens. Nevertheless, we found that TIE2 inhibition over a 2-week treatment period was more effective in reducing the growth of advanced *Pik3ca*-driven VMs in mice compared to rapamycin treatment. Our analysis did not reveal a statistically significant additive effect of BAY-826-mediated TIE2 inhibition when combined with rapamycin over a 2-week treatment period. However, in mice treated with soluble TIE2 from lesion initiation, short-term rapamycin administration after lesion establishment led to a significant reduction in vessel area and capillary SMC coverage compared to TIE2 ligand neutralization alone. While this treatment scheme is not therapeutically applicable to humans, it serves as proof of principle for the involvement of TIE2 in *Pik3ca*-driven VMs.

Rapamycin has additional context-dependent effects that warrant consideration. The well-known immunosuppressive effects of rapamycin^45^ may contribute to its beneficial effects in limiting vascular lesion growth, as has been proposed, at least in the case of lymphatic malformation^16^. While the second mTOR complex, the AKT-activating mTORC2, is not directly inhibited by rapamycin, prolonged treatment has been shown to affect its activity in certain cell types by inhibiting the assembly of the complex^46^. This likely explains the reduced AKT phosphorylation observed in cultured ECs carrying activating *TEK* or *PIK3CA* mutations after rapamycin treatment^47,48^. Conversely, in PI3K-dependent cancers, rapamycin treatment has been shown to trigger a feedback activation of PI3K–AKT, which involves mTORC1 substrates^46^. The context-dependent impact of rapamycin on mTOR-mediated AKT regulation may contribute to the varying success of rapamycin in different clinical contexts. Although AKT is acknowledged as the major downstream effector of PI3K, it should also be noted that PI3K is involved in other cellular processes independently of AKT. The complexity of the PI3K pathway underscores the potential for therapeutic interventions in VMs through inhibitors of its feedback regulators, such as TIE2. The critical role of TIE2 signaling in preserving vascular integrity may explain why its short-term inhibition is effective on advanced lesions, as opposed to rapamycin alone. However, it seems essential to target aberrant TIE2 activity without blocking it completely, to avoid potentially negative effects on vessel integrity. Interestingly, the compassionate use of the TIE2 kinase inhibitor rebastinib was reported to significantly improve both the outcome and quality of life of a patient with severe cervicofacial VMs caused by an activating *TEK* mutation, which had been refractory to other treatments^49^.

In summary, our findings highlight the importance of TIE2 signaling as a critical access point for treating advanced VMs. Even a short-term treatment regimen may offer an alternative strategy for patients with intractable lesions.

## Methods

### Mouse lines and treatments

*R26-LSL-Pik3ca*^*H1047R*^ mice^50^ were crossed with *Cdh5-CreER*^*T2*^ mice^51^ or *Vegfr1-CreER*^*T2*^ mice^16^, and analyzed on a C57BL/6J background. For clonal tracing, the mice were further crossed with *iChr2-Control-Mosaic*^27^ animals. Cre-mediated recombination was induced by topical application of 25 μg (*Cdh5-CreER*^*T2*^) or 50 μg (*Vegfr1-CreER*^*T2*^) of 4-OHT (Sigma-Aldrich, H7904), dissolved in acetone (5 mg ml^−1^), to the skin, equally distributed on both the dorsal and ventral sides of both ears. TIE2-inhibitor treatments were performed via oral gavage every second day with a dose of 25 mg per kg body weight of BAY-826 (Tocris Bioscience, 6579), which was dissolved in the emulsifying agent Kolliphor HS 15 (BASF, 70142-34-6) and prepared with a vehicle ratio of 50% H_2_O/40% Kolliphor/10% ethanol as previously described^52^. Rapamycin (LC Labs, R-5000), dissolved in dimethylsulfoxide, was administered via intraperitoneal injection at a dosage of 10 mg per kg body weight every second day. For combination treatments, BAY-826 and rapamycin were administered on alternate days. In all experiments, 4-OHT-treated Cre^−^ littermate mice carrying the *Pik3ca*^*H1047R*^ allele were used as controls. Cre^+^ vehicle controls were treated with either 50% H_2_O/40% Kolliphor/10% ethanol solution or dimethylsulfoxide.

### AAV vectors and treatments

For VEGF inhibition, we used AAV vectors encoding VEGF-Grab3-mFC (VEGF-Grab). Human VEGF-Grab3 insert was excised from the pDHFR-VEGF-Grab3 plasmid^19^ (a kind gift from H. M. Kim (Korea Advanced Institute of Science and Technology (KAIST); Graduate School of Medical Science & Engineering, KAIST, Daejeon, South Korea) and G. Y. Koh (Institute for Basic Science and KAIST, Daejeon) and cloned into a derivative shuttle vector of pUC19. The hVEGF-Grab3 was cut as a NheI-XbaI fragment and cloned into the destination vector psubCAG-WPRE. The mouse version, psubCAG-WPRE-mVEGF-Grab3, was created by site-directed mutagenesis and by replacing human sequences with the mouse paralogs synthesized by PCR from mouse cDNA. Like the original hVEGF-Grab3, the mVEGF-Grab3 also contains the CH2 and CH3 domains of the mouse IgG heavy chain, allowing for detection with anti-mouse IgG antibodies conjugated with horseradish peroxidase. As a control AAV9-mVEGFR3_4–7_-mFc (encoding a non-ligand-binding domain of VEGFR3 fused to Fc) was used. Recombinant AAV preparations of serotype 9 particles were generated as previously described^53^.

AAV9-VEGF-Grab3-mFC (VEGF-Grab) or AAV9-mVEGFR3_4-7_-mFc (control) in PBS was administered by intraperitoneal injection at a single dose of 1 × 10^11^ virus particles at 3 or 4 weeks of age. For TIE2 ligand neutralization, AAV9-mTIE2(ECD)-Flag^36^ at a single dose of 5 × 10^11^ virus particles in PBS was administered by intraperitoneal injection at 3 weeks of age. Experimental procedures on mice were approved by the Uppsala Animal Experiment Ethics Board (permit numbers 130/15, 5.8.18-06383/2020 and 5.8.18-03362/2021) and performed in compliance with all relevant Swedish regulations.

### Immunostaining of whole-mount ear skin

Ear skin was fixed in 4% paraformaldehyde at room temperature (RT) for 2 h and permeabilized in 0.3% Triton X-100 in PBS (PBST) for 10 min. After blocking with 5% bovine serum albumin (BSA) in PBST for 2 h, the tissues were incubated with primary antibodies overnight at 4 °C in 3% BSA-PBST blocking buffer. Subsequently, the tissues were washed with PBST and incubated with fluorescence-conjugated secondary antibodies for 2 h at RT under light protection. Proliferating cells were detected by labeling DNA synthesis using the Click-iT EdU Cell Proliferation Kit for Imaging (Thermo Fisher Scientific). In total, 25 mg per kg body weight of EdU was injected intraperitoneally 16 h before dissection of the ear skin. EdU staining was performed according to the manufacturer’s instructions with an incubation time of 40 min at RT. After additional washes in PBST, the samples were mounted in Fluoroshield (Sigma, F6182) histology mounting media and stored at 4 °C before imaging.

The following primary antibodies were used for whole-mount immunofluorescence (dilution 1:200): hamster anti-mouse CD31/PECAM1 2H8 (Invitrogen, MA3105), rat anti-mouse EMCN (Santa Cruz Biotechnology, sc-65495), goat anti-human SOX17 (R&D Systems, AF1924) and humanized monoclonal anti-ANGPT2 antibody (ABTAA)^54^, a kind gift from G. Y. Koh). Rabbit anti-ANGPT1 antibody (Proteintech, 23302-1-AP) was used at a dilution of 1:100. For αSMA staining a mouse Cy3-conjugated antibody (Sigma, clone 1A4, C6198) was used (dilution 1:500). Secondary antibodies used for whole-mount immunofluorescence were obtained from Jackson ImmunoResearch (dilution 1:500): donkey anti-rat IgG-AF488 (712-545-153), donkey anti-rat IgG-Cy3 (712-165-153), donkey anti-rat AF680 (712-625-153), donkey anti-goat AF680 (706-625-147), donkey anti-human Cy3 (709-165-149), rabbit anti-hamster-Cy3 (307-165-003) and donkey anti-rabbit AF488 (711-545-152).

### In situ PLA and immunostaining of paraffin sections

Mouse ear skin was fixed in 4% paraformaldehyde and embedded in paraffin. Tissues were sectioned to a thickness of 8 μm, and then subjected to deparaffinization and rehydration using xylene and a series of descending alcohol concentrations to remove paraffin. After rehydration, antigen retrieval was performed using 10 mM sodium citrate buffer, pH 6, at 95 °C. Sections were permeabilized with 0.3% PBST for 10 min at RT and blocked in 5% BSA-PBST for 1 h at 37 °C in a humidity chamber before immunostaining using rat anti-mouse EMCN (Santa Cruz Biotechnology, sc-65495) and rabbit anti‐mouse/human phospho‐Tie2 (pY992; R&D Systems, AF2720) antibodies (dilution 1:200). Alternatively, sections were subjected to PLA using rabbit pTyr (P-Tyr-1000 MultiMab, Cell Signaling, 8954, dilution 1:200) and goat anti-mouse TIE2 (R&D Systems, AF762, dilution 1:200), followed by co-staining using hamster anti-mouse CD31/PECAM1 2H8 (Invitrogen, MA3105, dilution 1:200). TIE2 and pTyr antibodies were used individually as technical controls. The PLA between TIE2 and pTyr was conducted following the NaveniFlex Tissue GR kit instructions with TEX615 detection fluorophore (Navinci, formerly Olink Bioscience, NT.GR.100.RED). Following primary antibody incubation or PLA reaction, sections were washed in TBST and subsequently incubated with donkey anti-rat IgG-AF488 (712-545-153, dilution 1:500) and donkey anti-rabbit IgG Highly Cross-Absorbed AF647 Plus (A32795, dilution 1:500) secondary antibodies for immunostaining, or rabbit anti-Syrian hamster secondary antibody AF594 (Jackson ImmunoResearch, 307-585-003, dilution 1:500) for PLA for 2 h at RT under light protection. After an additional washing step in TBST, sections were stained for DAPI (ready-made solution, Sigma-Aldrich, MBD0015) and slides were mounted using Eukitt Quick-hardening mounting media (Sigma-Aldrich, 03989). Human paraffin sections (5 µm) were treated as described above and antibodies used were rabbit pTyr (P-Tyr-1000 MultiMab, Cell Signaling, 8954, dilution 1:200) and goat anti-human TIE2 (R&D Systems, AF313, dilution 1:200). PLA dot quantification for human samples was automated using a custom script in Fiji, available at https://github.com/TMA-Lab/PIK3CA-driven-venous-malformations/. The study on human VMs was approved by the institutional review board of the University of Freiburg, Germany (21-1200) and of University of Louvain, Brussels, Belgium (B403201629786). Informed consent was obtained from the participants (adults) or from the participants and their parents (children). Analysis of human biopsy material was approved by the Swedish Ethical Review Authority (Etikprövningsmyndigheten, Dnr 2020-00987).

### Intravital microscopy

Mice undergoing intravital imaging received an intravenous injection of 25 µg of Alexa Fluor 647-labeled, non-blocking PECAM1 antibody (mouse, clone 390, 102416, BioLegend) via tail vein injection. The antibody was dissolved in 100 µl of sterile saline. Mice were anesthetized with an intraperitoneal injection of ketamine (100 mg per kg body weight) and xylazine (12.5 mg per kg body weight), both dissolved in sterile saline. The dorsal ear skin was secured onto a custom-made, 3D-printed stage for imaging. During the imaging session, animals received an eye lubricant and thermal support. After the session, mice were rehydrated with an intraperitoneal saline injection. Imaging was performed using a Leica SP8 DIVE platform equipped with a Ti:Sapphire multiphoton laser, capable of emitting a tunable range of 680–1,300 nm and a fixed 1,045-nm laser line. A water-immersion HC IRAPO ×25/1.0 objective was used for imaging. For longitudinal studies, the PECAM1 antibody was re-injected, and the region of interest was relocated in subsequent imaging sessions with the LAS X integrated navigator function, using the vascular anatomy for orientation.

### Immunofluorescence and H&E staining of cryosections

Mouse female reproductive tracts were fixed in 4% paraformaldehyde overnight. To ensure cryoprotection, the tissues were fist incubated in 15% sucrose–PBS solution, followed by 30% sucrose overnight, and embedded in optimal cutting temperature compound on dry ice. Cryosections of 10–20-μm thickness were then obtained using a cryostat and collected on glass slides, which were stored at −80 °C. Following thawing and rehydration, immunostaining of the cryosections was performed as described for whole-mount immunostaining. Additionally, adjacent sections were subjected to H&E staining. Before mounting with Eukitt Quick-hardening mounting medium, the H&E-stained sections underwent dehydration and xylene treatment as described earlier.

### Immunofluorescence staining of the thyroid gland

Mouse thyroid glands were dissected without additional cleaning from the connective tissue holding the gland and fixed in 4% paraformaldehyde overnight at 4 °C. The tissue was permeabilized in blocking solution (3% BSA, 0.5% fetal bovine serum (FBS) in PBST) overnight before primary antibodies were added for 2 days, followed by washing and staining with secondary antibodies. After staining, muscle and connective tissue covering the gland were dissected without damaging the vasculature underneath, followed by incubation and mounting in RIMS clearing imaging media (refractive index = 1.46).

### Confocal microscopy and image quantification

A Leica SP8 or a Leica STELLARIS 5 confocal microscope with a white light laser and ×10/0.45 C-Apochromat (HC PL APO CS2), ×20/0.75 (HC PL APO CS2), ×25/0.95 (HC FLUOTAR L VISIR) or ×63/1.20 (HC PL APO) objectives and Leica Application Suit X software were used to acquire confocal images. Maximum intensity projection of *z*-stacks was generated to represent the entire blood vessel network, or the entire tissue section (for paraffin and cryosections). A Leica Thunder Imaging System was used for tile scans in Fig. 8b. Brightfield images shown in Fig. 8a and Extended Data Figs. 1b,c and 5a–c were obtained using a Leica DMi8 microscope. Images were processed and analyzed using Fiji ImageJ software (National Institutes of Health, version 2.14.0) and imported into Adobe Illustrator for figure panels. For vessel area quantification, images were thresholded and converted into binary images, followed by segmentation and calculation of the percentage of EMCN^+^ vessel area of the total ear skin area. In some cases, measurements from both ears were taken, and the average value was calculated for an individual mouse. Vessel diameter was measured by segmenting veins into 50-µm intervals along their length. At each 50-µm segment, the diameter was measured using Fiji, and the average diameter was calculated for each vessel. To assess SMC coverage in human tissue sections, veins were segmented at 15-µm intervals, and the width of the αSMA^+^ cell layer was measured using Fiji software. SMC coverage was additionally analyzed by identifying SMC nuclei (DAPI stained). Regions containing more than two rows of αSMA-associated nuclei were classified as multilayered. All calculations were performed in Microsoft Excel.

### Analysis of mouse scRNA-seq data

Smart-Seq2 RNA-seq of dermal ECs isolated from the ear skin of 4-OHT-treated 5-week-old *Pik3ca*^*H1047R*^*;Cdh5-CreER*^*T2*^ mice (*n* = 5) and Cre^−^ littermate control mice (*n* = 2) of mixed genders, as well as one wild-type C57BL/6J mouse, not treated with 4-OHT, was performed previously^16^. Preprocessed data deposited in the Gene Expression Omnibus (GEO) under accession number GSE201916 were analyzed further in RStudio (desktop version 2021.09.2 + 382 to 2023.09.1) using R (versions 4.0.3 to 4.3.2) with the Seurat package (versions 3.1.1 to 5.1.0)^55–57^. As previously described^16^, raw expression data analysis of combined control and mutant mice BECs included normalization, Harmony batch correction (version 1.0)^58^ and integration, Louvain graph-based clustering, nonlinear dimensional reduction and visualization using UMAP. Cluster markers were detected by performing a DEG analysis (Wilcoxon rank-sum test, marker genes selected by *P* value with Bonferroni correction and logarithmic fold change > 0.25). One cluster of contaminating immune cells was identified based on top cluster markers including Clec5a, Dok2 and Cd86, and removed before further downstream analysis. Count data of 350 control BECs, which include Cre^−^ littermates and wild-type C57BL/6J, were extracted from the combined dataset and processed following the same steps as described above, but using canonical correlation analysis for batch effect correction instead. An additional 29 cells mainly characterized by ribosomal and mitochondrial reads were removed.

### GO term enrichment analysis

To identify overrepresented GO terms within a DEG list, the GOstats package was used (version 2.56.0). Gene sets with 5–1,000 genes were considered for analysis, and significant pathways were identified based on a *P*-value threshold < 0.00001 and gene count/term > 10. The relevance of GO terms was further assessed by sorting them based on their odds ratios, calculated as the ratio of a GO term’s occurrence in the DEG list to its occurrence in a universal gene list obtained from org.Mm.eg.db (version 3.16.0). Selected top pathways were visualized using ggplot2 (version 3.4.2).

### Transcription factor prediction and kinase enrichment analysis

Transcription factor prediction analysis was performed using the TFactS (Transcription Factor Prediction using ChIP–seq, version 0.99.0)^59^ tool using default parameters. DEG analysis results (threshold logarithmic fold change > 1), including upregulated and downregulated genes and associated expression values, were prepared as input data. Kinase enrichment analysis was performed using TFactS transcription factor prediction results as an input for Kinase Enrichment Analysis 3 (version 3) using default parameters^60^.

### Trajectory inference and interaction analysis

Trajectory inference analysis was conducted to capture gradual phenotypic transitions across clusters based on gene expression profiles. For the control dataset, we utilized the SCORPIUS algorithm (version 1.0.5)^61^ (Extended Data Fig. 2a,b), which uses a graph-based and feature selection strategy (with *k* = 5) to order cells and clusters along the trajectory. For the combined dataset of *Pik3ca*^*H1047R*^ and control BECs, we used SLINGSHOT (version 2.2.0)^62^ (Fig. 3 and Extended Data Fig. 3a) for unsupervised trajectory analysis. SLINGSHOT utilized a minimum spanning tree (MST) construction to define branch points along the trajectory, to allow visualizing the divergence of cell fates. Cells were ordered based on pseudotime values, representing gradual gene expression changes along the trajectory. The algorithm automatically selected the root cell based on the fewest overall cluster connections, providing a starting point for the trajectory. SCORPIUS was subsequently used to analyze the separate control and *Pik3ca*^*H1047R*^ mutant trajectories using default parameters (Fig. 3a,d and Extended Data Figs. 2a,b and 3a). Genes of importance were visualized in a heat map, ranked according to their position along the trajectory. CellChat^63^ was used for signaling prediction between EC subtypes and visualization, using default parameters.

### Analysis of human scRNA-seq data

EC scRNA-seq expression data were obtained from five publications^64–68^, comprising 25 individual patient samples. After merging datasets, cells expressing fewer than 200 genes were filtered out. Mitochondrial genes, genes expressed in fewer than three cells, and potential doublets were removed, along with counts of MALAT1, a long noncoding RNA known to cause bias in scRNA-seq analysis. To integrate the data from multiple samples, we first normalized the expression within each sample using log normalization and identified the top 2,000 highly variable features using the variance-stabilizing transformation method. Integration features were then selected, and integration anchors were identified to facilitate data integration across all samples. Further clustering and downstream analysis were performed following the same approach as that used for mouse scRNA-seq analysis.

For gene orthology conversion between mouse and human, the ‘HOM_MouseHumanSequence’ dataset from the Mouse Genome Database (MGD) of the Jackson Laboratory was used^69^. Mouse genes were mapped to human orthologs using a custom script available at https://github.com/TMA-Lab/PIK3CA-driven-venous-malformations/. Mouse genes without identifiable orthologs were excluded from the analysis. The comparative analysis of conserved genes between the two species was visualized in a Venn diagram. The proportion of conserved genes was calculated, along with a list of non-conserved genes for each species.

### Cells and cell culture

Pooled HUVECs from different donors were purchased from Lonza (CC-2519) and cultured in endothelial basal medium (EBM, Lonza) supplemented with hydrocortisone (1 µg ml^−1^), bovine brain extract (12 µg ml^−1^), gentamicin (50 µg ml^−1^), amphotericin B (50 ng ml^−1^), human recombinant epidermal growth factor (10 ng ml^−1^) and 10% FBS (Life Technologies). Human embryonic kidney cells (HEK293FT) were purchased from Life Technologies (R70007) and cultured in DMEM supplemented with 10% FBS (Life Technologies) and geneticin (500 µg ml^−1^; Invitrogen). Cells were tested negative for mycoplasma and maintained at 37 °C in a humidified atmosphere with 5% CO_2_. Authentication of cell lines was based on morphology and immunohistochemistry profile.

To stimulate TIE2 phosphorylation, rhANGPT1 (R&D Systems, 923-AN-025/CF) was reconstituted in PBS and used at the concentrations of 50 ng ml^−1^ (PIK3CAH1047R expression, Fig. 5e) or 200 ng ml^−1^ (FOXO1^A3^ expression, Fig. 5f) for 30 min. To inhibit TIE2 phosphorylation, HUVECs were pretreated with 1 µg ml^−1^ BAY-826 (Tocris, 6579) for 10 min before rhANGPT1 stimulation.

### Adenoviral transductions

Sub-confluent HUVECs were transduced with custom-made adenoviruses (Vector Biolabs) to overexpress the FLAG-tagged human FOXO1^A3^ that cannot be inhibited by PI3K–AKT signaling and is, thus, constitutively nuclear. Adenoviruses that contain an empty cytomegalovirus promoter (AdCtrl, Vector Biolabs, 1300) were used as a control. For transductions, HUVECs were incubated in EBM (Lonza) containing 0.1% (vol/vol) BSA (Sigma, 1595) for 4 h and transduced with control or FOXO1^A3^ for an additional 4 h in the presence of 8 µg ml^−1^ polybrene (Santa Cruz Biotechnology). Afterwards, HUVECs were washed five times with Hank’s Balanced Salt Solution (Life Technologies) and cultured in EBM with 10% FBS and supplements.

### ChIP

ChIP studies were performed as described in ref. ^29^. Briefly, HUVECs were fixed with 1% formaldehyde for 15 min and quenched with 0.125 M glycine. Chromatin was isolated by the addition of lysis buffer, followed by disruption with a Dounce homogenizer. Lysates were sonicated and the DNA sheared to an average length of 300–500 bp. Genomic DNA (input) was prepared by treating aliquots of chromatin with RNase, proteinase K and heat for reverse crosslinking, followed by ethanol precipitation. Pellets were resuspended and the resulting DNA was quantified on a NanoDrop spectrophotometer. Chromatin yield was extrapolated from the total volume. Sheared chromatin (30 μg) was precleared with protein A agarose beads (Invitrogen). Genomic DNA regions of interest were isolated using 4 μg of ChIP-grade antibodies against FOXO1 (rabbit, Abcam, ab39670), H3K4me3 (mouse, Active Motif, 39159) and H3K27ac (rabbit, Active Motif, 39133). Complexes were washed, eluted from the beads with SDS buffer and subjected to RNase and proteinase K treatment. Crosslinks were reversed by incubation overnight at 65 °C, and ChIP DNAs were purified by phenol–chloroform extraction and ethanol precipitation.

### ChIP–seq

Illumina sequencing libraries were prepared from ChIP and input DNAs by the standard consecutive enzymatic steps of end-polishing, dA-addition and adaptor ligation. After a final PCR amplification step, the resulting DNA libraries were quantified and sequenced on Illumina’s NextSeq 500 (75-nucleotide reads, single end). Reads were aligned to the human genome (hg38) using the BWA algorithm^70^ (default settings). Duplicate reads were removed and only uniquely mapped reads (mapping quality ≥ 25) were used for further analysis. Alignments were extended in silico at their 3′-ends to a length of 200 bp, which is the average genomic fragment length in the size-selected library, and assigned to 32-nucleotide bins along the genome. The resulting histograms (genomic ‘signal maps’) were stored in bigWig files. Peak locations were determined using the MACS algorithm (version 2.1.0)^71^ with a *P*-value cutoff = 1 × 10^−7^. Peaks that were on the ENCODE blacklist of known false ChIP–seq peaks were removed. Signal maps and peak locations were used as input data to the Active Motifs proprietary analysis program, which creates tables containing detailed information on sample comparison, peak metrics, peak locations and gene annotations. Binding motifs were identified with the findMotifsGenome program of the HOMER package (version 4.10.4)^72^ using default parameters and input sequences comprising ±100 bp from the center of the top 1,000 peaks. Individual profiles were produced with a window of 5 bp. All profiles were plotted on a normalized reads-per-million basis. The processed data were plotted and visualized using software of the R project for statistical computing.

### ATAC-seq

For ATAC-seq, transduced HUVECs were freshly processed. In brief, 50,000 cells were centrifuged at 500*g* for 5 min at 4 °C and washed with PBS. The cell pellet was resuspended in 50 µl lysis/transposition reaction mix (12.5 µl THS-TD-Buffer, 2.5 µl Tn5, 5 µl 0.1% digitonin and 30 µl water) and incubated at 37 °C for 30 min with occasional snap mixing. Purification of the DNA fragments was then done using the MinElute PCR Purification Kit (Qiagen). Amplification of the library together with indexing primers was performed as described elsewhere^73^. Libraries were mixed in equimolar ratios and sequenced on the NextSeq 500 platform using V2 chemistry and a paired-end setup. Raw reads were trimmed according to RNA-seq reads and were aligned versus the human genome version hg38 (Ensembl release 101) using STAR (version 2.7.9a) with the parameters ‘--outFilterMismatchNoverLmax 0.1 --outFilterMatchNmin 20 --alignIntronMax 1 --alignSJDBoverhangMin 999 --outFilterMultimapNmax 1 --alignEndsProtrude 10 ConcordantPair’^74^ and retaining only unique alignments to exclude reads of uncertain origin. Reads were further deduplicated using Picard (version 2.25.5) to mitigate PCR artifacts leading to multiple copies of the same original fragment. Reads aligned to the mitochondrial chromosome were removed. The Macs peak caller (version 3.0.0a6) was used to accommodate for the range of peak widths typically expected for ATAC-seq^71^. The minimum *q* value was set to −4, and the false discovery rate was changed to 0.0001. Peaks overlapping ENCODE blacklisted regions (known misassemblies, satellite repeats) were excluded. To enable the comparison of peaks in different samples to assess reproducibility, the resulting lists of significant peaks were overlapped and unified to represent identical regions. Sample counts for union peaks were produced using bigWigAverageOverBed (UCSC Toolkit version 4) and normalized with DESeq2 (version 1.30.1) to compensate for differences in sequencing depth, library composition and ATAC-seq efficiency^75^. Peaks were annotated with the promoter of the nearest gene in range (transcription start site ± 5,000 nucleotides) based on reference data of GENCODE (version M15).

### Lentivirus generation and transductions

For doxycycline-inducible lentiviral expression of mutant PIKCA^H1047R^ or constitutively nuclear FOXO1 (FOXO1^A3^) used in Fig. 6e, f, V5-tagged *PIKCA*^*H1047R*^ or *FOXO1*^*A3*^ cDNAs were cloned into pLVX-TetOne-Puro (Clontech). Lentivirus production was performed by co-transfection of HEK293FT cells with pMD2.G (Addgene, 12259), psPAX2 (Addgene, 12260) and transfer plasmids. Transfections were carried out using Lipofectamine 2000 transfection reagent (Life Technologies), as previously described^29^. Viruses were collected 48 h and 72 h after transfection and filtered through a 0.45-μm filter. HUVECs were transduced with lentiviruses for 16 h in the presence of 8 μg ml^−1^ polybrene (Santa Cruz) and selected with 1 μg ml^−1^ puromycin (InvivoGen, ant-pr-1). Lentiviral-mediated transgene expression was induced with 400 ng ml^−1^ doxycycline (Sigma, D9891) for 48 h before sample collection.

### Immunoprecipitation

Immunoprecipitation and analysis of TIE2 phosphorylation were performed as previously described^76^. Cells overexpressing PIK3CA^H1047R^ or FOXO1^A3^ mutants were starved of serum for 6 h in EBM medium (Lonza) containing 0.1% (vol/vol) BSA (Sigma, 1595) before the indicated treatments and rhANGPT1 stimulation. HUVECs were lysed in immunoprecipitation buffer (10 mM Tris-Cl pH 7.4, 150 mM NaCl, 5 mM EDTA, 10% glycerol and 1% Triton X-100) freshly supplemented with 1× protease/phosphatase inhibitor cocktail (Cell Signaling Technology, 5872) and 1 mM phenylmethylsulfonyl fluoride. Cell lysates were cleared by centrifugation at 13,800*g* for 15 min at 4 °C, and protein concentrations were determined by the Bradford method. Equal amounts of total protein lysates were incubated overnight at 4 °C with 1 μg of goat anti-human anti-TIE2 antibody (R&D Systems, AF313), and immunoprecipitations were performed with magnetic Protein G Dynabeads (Invitrogen, 10-003-D) for 2 h. Beads were washed three times with immunoprecipitation buffer and proteins were eluted by incubation at 95 °C for 10 min with 2× SDS sample buffer before immunoblotting analysis.

### Western blot analysis and antibodies

Proteins were resolved by SDS–PAGE using Criterion TGX Precast gels (Bio-Rad) and transferred onto nitrocellulose membranes using the Trans Turbo Blot system (Bio-Rad). Membranes were blocked in TBS buffer containing 5% (wt/vol) BSA or 5% (wt/vol) milk and 0.01% (vol/vol) Tween-20 for 1 h at RT. Primary antibodies diluted in blocking buffer were incubated overnight at 4 °C. Peroxidase-conjugated secondary antibodies were incubated for 2 h at RT. Immunoblots were visualized using Clarity Western ECL kit (Bio-Rad) and the ChemiDoc MP Imaging System (Bio-Rad). Primary anti-human antibodies were obtained from Cell Signaling Technologies and used at the following dilutions: ANGPT2 (D200; rabbit, 50697), pan-AKT (rabbit, 4691), phospho-AKT (D9E; rabbit, Ser473, 4060) and phospho-TYR (mouse, 96215) at a dilution of 1:1,000; phospho-S6 ribosomal protein (rabbit, Ser235/236, 4857) and S6 ribosomal protein (5G10; rabbit, 2217) at a dilution of 1:5,000; α/β-tubulin (rabbit, 2148) at a dilution of 1:5,000; and V5-tag (D3H8Q; rabbit, 13202) at a dilution of 1:2,500. Anti-TIE2 antibody (goat, AF313) was obtained from R&D Systems and used at a dilution of 1:1,000. Horseradish peroxidase-conjugated secondary antibodies from Jackson ImmunoResearch were applied at a dilution of 1:5,000, including goat anti-rabbit (111-035-008), rabbit anti-mouse (315-035-003) and rabbit anti-goat (305-036-008).

### Statistics and reproducibility

GraphPad Prism 10 software was utilized for statistical analysis and graphical representation of the data. For comparisons involving multiple groups, one-way ANOVA was applied, followed by Tukey’s multiple-comparison test. Data between two groups were compared with unpaired or paired two-tailed Student’s *t*-test assuming equal variance. When the data were not normally distributed, a Mann–Whitney *U*-test was used instead. Differences were considered statistically significant when the adjusted *P* value was below the predetermined threshold: ****P < 0.0001, ***P < 0.001, **P < 0.01, *P < 0.05; NS, *P* > 0.05. The specific methods used for multiple-testing corrections are described in the respective figure legends. For GO term analysis using GOstats, a standard hypergeometric tests was performed to assess the enrichment of GO terms within specific DEG lists. The GO term sizes were set to contain a minimum of 5 and a maximum of 1,000 members of the mouse genome. Cluster markers from scRNA-seq data were identified using the Wilcoxon rank-sum test, and marker genes were selected based on the adjusted *P* value with Bonferroni correction and logarithmic fold change as indicated. Data with *N* ≥ 2 were independently replicated in separate experiments, using mice from at least two different litters.

### Reporting summary

Further information on research design is available in the Nature Portfolio Reporting Summary linked to this article.

## Supplementary information

Reporting Summary

Supplementary Table 1 Clinical features of individuals with VMs associated with *PIK3CA* or *TEK* mutations.

## Source data

Source Data Fig. 1 Statistical source data for Fig. 1d,f.

Source Data Fig. 2 Statistical source data for Fig. 2b,c,e–g.

Source Data Fig. 3 Statistical source data for Fig. 3d,e.

Source Data Fig. 4 Statistical source data for Fig. 4a–c,h.

Source Data Fig. 5 Statistical source data for Fig. 5a,c.

Source Data Fig. 6 Statistical source data for Fig. 6b,d,f,i.

Source Data Fig. 7 Statistical source data for Fig. 7c–e.

Source Data Fig. 8 Statistical source data for Fig. 8c,d,g,h,k.

Source Data Extended Data Fig. 1 Statistical source data for Extended Data Fig. 1e.

Source Data Extended Data Fig. 2 Statistical source data for Extended Data Fig. 2f.

Source Data Extended Data Fig. 3 Statistical source data for Extended Data Fig. 3a–c.

Source Data Extended Data Fig. 4 Statistical source data for Extended Data Fig. 4a–d.

Source Data Extended Data Fig. 7 Statistical source data for Extended Data Fig. 7a.

Source Data Extended Data Fig. 8 Statistical source data for Extended Data Fig. 8c–h.

Source Data Extended Data Fig. 9 Statistical source data for Extended Data Fig 9a,c,d,f.

Source Data Unprocessed Gels Unprocessed western blots for Fig. 5e,f.

## Data Availability

Source data are provided with this paper. All other data supporting the findings of this study are available within the paper and its Supplementary Information. Any additional information required to interpret, replicate or build upon the findings of this study are available from the corresponding author upon reasonable request. ChIP–seq and bulk RNA-seq data have been deposited in the GEO under accession codes GSE201916 and GSE128636. Mouse and human dermal BEC data are available at https://makinenlab.shinyapps.io/Mouse_DermalBloodEndothelialCells/ and https://makinenlab.shinyapps.io/Human_DermalBloodEndothelialCells/, respectively, generated using ShinyCell, a Shiny package of Rstudio (https://shiny.rstudio.com/). Reference data and libraries used for data analysis are the human genome version hg38 (Ensembl release 101; http://aug2020.archive.ensembl.org/Homo_sapiens/Info/Index/ and http://aug2020.archive.ensembl.org/Homo_sapiens/Info/Index/), GENCODE (version M15; https://www.gencodegenes.org/mouse/release_M15.html) and Kinase Enrichment Analysis 3 (version 3) libraries (https://maayanlab.cloud/kea3/templates/libraries.jsp).

## Code availability

Scripts used for the analyses presented in this study are available on GitHub via https://github.com/TMA-Lab and https://github.com/TMA-Lab/PIK3CA-driven-venous-malformations/. Correspondence and requests for materials should be addressed to T.M.
