# Supplementary material for: Angiopoietin–TIE2 feedforward circuit promotes PIK3CA-driven venous malformations
Source: Nat Cardiovasc Res. 2025 May 23;4(7):801–20. doi: 10.1038/s44161-025-00655-9 (PMC12259471; doi:10.1038/s44161-025-00655-9)
Supplement: Supplementary file 1 — Reporting Summary [file 44161_2025_655_MOESM1_ESM.pdf]

nature portfolio

Corresponding author(s): Mäkinen, T

Last updated by author(s): Apr 3, 2025

Reporting Summary

Nature Portfolio wishes to improve the reproducibility of the work that we publish. This form provides structure for consistency and transparency in reporting. For further information on Nature Portfolio policies, see our Editorial Policies and the Editorial Policy Checklist.

Statistics

For all statistical analyses, confirm that the following items are present in the figure legend, table legend, main text, or Methods section.

n/a	Confirmed	
☐	☒	The exact sample size (n) for each experimental group/condition, given as a discrete number and unit of measurement
☐	☒	A statement on whether measurements were taken from distinct samples or whether the same sample was measured repeatedly
☐	☒	The statistical test(s) used AND whether they are one- or two-sided<br>*Only common tests should be described solely by name; describe more complex techniques in the Methods section.*
☒	☐	A description of all covariates tested
☐	☒	A description of any assumptions or corrections, such as tests of normality and adjustment for multiple comparisons
☐	☒	A full description of the statistical parameters including central tendency (e.g. means) or other basic estimates (e.g. regression coefficient) AND variation (e.g. standard deviation) or associated estimates of uncertainty (e.g. confidence intervals)
☐	☒	For null hypothesis testing, the test statistic (e.g. F, t, r) with confidence intervals, effect sizes, degrees of freedom and P value noted<br>*Give P values as exact values whenever suitable.*
☒	☐	For Bayesian analysis, information on the choice of priors and Markov chain Monte Carlo settings
☒	☐	For hierarchical and complex designs, identification of the appropriate level for tests and full reporting of outcomes
☒	☐	Estimates of effect sizes (e.g. Cohen's d, Pearson's r), indicating how they were calculated

Our web collection on statistics for biologists contains articles on many of the points above.

Software and code

Policy information about availability of computer code

Data collection	Leica Application Suite X (Version 4.5.0.25531 and earlier) [image aquisition]<br>Illumina NextSeq 500 [RNA-, ATAC- and ChIP-seq]

Data analysis	Image J (Version 2.14.0/1.54f or earlier) [image processing and analysis]<br>Adobe Illustrator (Version 27.7 or earlier) [illustration]<br>Microsoft Excel (2019, Version 16.74) [numeric data processing and organisation]<br>Graphpad Prism 10 [numeric data analysis and visualization]<br>R for Mac OS (Version 4.0.3 to 4.3.2 or earlier), Rstudio (Version 2021.09.2 + 382 to 2023.09.1), Seurat (Version 3.1.1 to 5.1.0), GOstats (v2.56.0), org.Mm.eg.db (version 3.16.0),ggplot2 (version 3.4.2),SLINGSHOT (version 2.2.0), SCORPIUS algorithm (version 1.0.5) [scRNA-seq data processing, analysis and visualization]<br>TFactS (TFactSR v0.99.0) [data analysis]<br>Kinase Enrichment Analysis 3 (KEA3, version 3) [data analysis]<br>shinyapps.io by Posit [data sharing platform]<br>MACS (v2.1.0), BWA (v0.7.12); bcl2fastq2 (v2.20); Samtools (v0.1.19); BEDtools (v2.25.0); wigToBigWig (v4), HOMER(Version 4.10),STAR (v2.7.9a),human genome version hg38 (Ensembl release 101), Picard (v2.25.5), Macs peak caller (v3.0.0a6), DESeq2 (v1.30.1), reference data of GENCODE (vM15) [RNA-,AATAC- and ChIP-seq]<br><br>custom codes developed in the study:<br>https://github.com/TMA-Lab/PIK3CA-driven-venous-malformations

For manuscripts utilizing custom algorithms or software that are central to the research but not yet described in published literature, software must be made available to editors and reviewers. We strongly encourage code deposition in a community repository (e.g. GitHub). See the Nature Portfolio guidelines for submitting code & software for further information.

Data

Policy information about availability of data

All manuscripts must include a data availability statement. This statement should provide the following information, where applicable:

- Accession codes, unique identifiers, or web links for publicly available datasets
- A description of any restrictions on data availability
- For clinical datasets or third party data, please ensure that the statement adheres to our policy

All data supporting the findings of this study are available within the paper and its supplementary information files. All data supporting the quantitative findings of this study are provided as source data. Any additional information required to interpret, replicate or build upon the findings of this study are available from the corresponding author upon reasonable request.

ChIP-seq and bulk RNA–seq data have been deposited in the GEO under accession code GSE128636.

The data on mouse and human dermal BECs are available at the following searchable web applications, generated using ShinyCell an shiny package of Rstudio (https://shiny.rstudio.com):
Mouse BECs: https://makinenlab.shinyapps.io/Mouse_DermalBloodEndothelialCells/
Human BECs: https://makinenlab.shinyapps.io/Human_DermalBloodEndothelialCells/

GEO accession numbers for published RNA-seq data: GSE201916, GSE128636.
https://www.ncbi.nlm.nih.gov/geo/query/acc.cgi?acc=GSE201916
https://www.ncbi.nlm.nih.gov/geo/query/acc.cgi?acc=GSE128636

Reference data and libraries used for data analysis:
human genome version hg38 (Ensembl release 101): http://aug2020.archive.ensembl.org/Homo_sapiens/Info/Index
reference data of GENCODE (vM15): https://www.gencodegenes.org/mouse/release_M15.html
Kinase Enrichment Analysis 3 (KEA3, version 3) libraries: https://maayanlab.cloud/kea3/templates/libraries.jsp

Research involving human participants, their data, or biological material

Policy information about studies with human participants or human data. See also policy information about sex, gender (identity/presentation), and sexual orientation and race, ethnicity and racism.

Reporting on sex and gender	7 females and 8 males were included in the study. Details on sex are provided in Supplementary Table 1.
Reporting on race, ethnicity, or other socially relevant groupings	n/a
Population characteristics	Clinical features of VM patients with PIK3CA or TEK mutation(s) included in the study are summarized in Supplementary Table 1. Age of the patients: 2-41 years (PIK3CA mutation, n=7), 11-36 years (TEK mutation, n=6). Normal skin as a control: 60 and 68 years (n=2).
Recruitment	Residual tissue of VM was collected from patients undergoing therapeutic surgery. The decision to pursue surgery as a therapeutic option had been made by the attending physician based on clinical evaluation. When surgery is performed, the resected tissues are routinely screened for TEK and PIK3CA mutations. We specifically looked for cutaneous lesions, of which 7 representative lesions with TEK mutations and 6 with PIK3CA mutations were selected for this study. There is no identifiable bias with the approach.
Ethics oversight	The study protocol was approved by the ethical committees of the University of Freiburg, Germany, and the Medical Faculty

Ethics oversight	of the University of Louvain, Brussels, Belgium. Analysis of human VM biopsy material was approved by Swedish Ethical Review Authority (Etikprövningsmyndigheten).

Note that full information on the approval of the study protocol must also be provided in the manuscript.

Field-specific reporting

Please select the one below that is the best fit for your research. If you are not sure, read the appropriate sections before making your selection.

☒ Life sciences ☐ Behavioural & social sciences ☐ Ecological, evolutionary & environmental sciences

For a reference copy of the document with all sections, see nature.com/documents/nr-reporting-summary-flat.pdf

Life sciences study design

All studies must disclose on these points even when the disclosure is negative.

Sample size	Data are based on a minimum of three biological replicates per condition (except for Fig. 1f and ED Fig. 2d n=2 for some groups). Sample size (n) is indicated in the figure legends and source data. No statistical methods were used to predetermine sample size; the chosen sample sizes were based on ethical consideration and sufficient to capture biologically relevant differences.
Data exclusions	Data exclusion in scRNA-seq analysis is based on established quality control criteria, and documented in the main text.
Replication	Experimental repetition (N) are indicated in the source data. Data presented in Fig. 1d,f; 4h; 5c; 6b and Extended Data Fig. 9f were obtained from one experiment. All other data were successfully replicated in independent experiments and with mice from at least 2 separate litters.
Randomization	Mice were randomly allocated into experimental (treatment) groups based on their genotypes. Lttermates were included as controls and randomly allocated into experimental (treatment) groups. When applicable, mice from different cages, but within the same experimental group, were selected to ensure randomization. Both female and male mice were included in analyses. Data were collected from different litters on different days and experiments were performed for different batches at different time points.
Blinding	Blinding was used for human sample quantifications performed before mutation information was provided by the clinics. Blinding was used for RNA-seq, ATAC-seq and ChIP-Seq GSE128636 data collection and analysis, performed by participants (blinded to group allocation) other than the experimental designer. For other analyses, because the same investigator designed and conducted the experiments, full blinding was not feasible. However, objective measurements and automated analyses were used where possible to minimize potential bias.

Reporting for specific materials, systems and methods

We require information from authors about some types of materials, experimental systems and methods used in many studies. Here, indicate whether each material, system or method listed is relevant to your study. If you are not sure if a list item applies to your research, read the appropriate section before selecting a response.

Materials & experimental systems

n/a	Involved in the study	
☐	☒	Antibodies
☐	☒	Eukaryotic cell lines
☒	☐	Palaeontology and archaeology
☐	☒	Animals and other organisms
☒	☐	Clinical data
☒	☐	Dual use research of concern
☒	☐	Plants

Methods

n/a	Involved in the study	
☐	☒	ChIP-seq
☒	☐	Flow cytometry
☒	☐	MRI-based neuroimaging

Antibodies

Antibodies used

Primary antibodies used for whole mount immunofluorescence (dilution 1:200 if not stated differently):

Hamster anti-mouse PECAM1/CD31 2H8 (Invitrogen, #MA3105, LOT: UJ290337),
514 product citations:https://www.rndsystems.com/products/human-mouse-rat-cd31-pecam-1-antibody_af3628#product-citations

Rat anti-mouse EMCN (Santa Cruz Biotechnology, #sc-65495, LOT: #K1121),
423 product citations: https://www.scbt.com/p/endomucin-antibody-v-7c7?srsltid=AfmBOooCiv7s_uiGRWCsnTg4HHaGsuqfJeLNDpIzE1_TeaKpRjhPfQyI#citations

Goat anti-human SOX17 (R&D Systems, #AF1924, LOT: #KGA1022031),
500 product citations: https://www.rndsystems.com/products/human-sox17-antibody_af1924?gclsrc=aw.ds&gad_source=1&gbraid=0AAAAAD_kmX0NBr7Lc1CuFZ9AcPaNc4_Sk&gclid=Cj0KCQjwna6_BhCbARIsALId2Z1nMIvmSVYM7G19geLujzRmBR9o1xQb0KGnAvBeTXn5SjI2x3Z9ijAaAspfEALw_wcB#product-citations

Humanized monoclonal anti-ANGPT2 antibody (ABTAA, provided by Gou Young Koh and not commercially available).
Validated in: Han, S. et al. Amelioration of sepsis by TIE2 activation–induced vascular protection. Sci. Transl. Med. 8, 335ra55-335ra55 (2016).

Rabbit anti-ANGPT1 antibody (Proteintech, #23302-1-AP, dilution 1:100).
28 product citations:https://www.ptglab.com/products/ANGPT1-Antibody-23302-1-AP.htm?srsltid=AfmBOopremYo_fnYLeUHEoJ_3HpVbIKUn_l5L8a-PGfPpSWfFfylaypl

Secondary antibodies used for whole mount immunofluorescence (dilution 1:500):

Donkey anti-rat IgG-AF488 (JIR, 712-545-153, LOT: #156685),
174 product citations: https://www.jacksonimmuno.com/catalog/products/712-545-153

Donkey anti-rat IgG-Cy3 (JIR, 712-165-153, LOT: #145787),
422 product citations: https://www.jacksonimmuno.com/catalog/products/712-165-153

Donkey anti-rat AF680 (JIR, 712-625-153, LOT: #156733),
4 product citations: https://www.jacksonimmuno.com/catalog/products/712-625-153

Donkey anti-goat AF680 (JIR, 705-625-147, LOT: #147752),
15 product citations: https://www.jacksonimmuno.com/catalog/products/705-625-147

Donkey anti-human Cy3 (JIR, 709-165-149, LOT: #139754),
21 product citations: https://www.jacksonimmuno.com/catalog/products/709-165-149

Rabbit-anti-hamster-Cy3 (JIR, 307-165-003, LOT: #120313).
2 product citations:https://www.jacksonimmuno.com/catalog/products/307-165-003

Conjugated antibodies used for whole mount immunofluorescence (dilution 1:500):

Mouse anti-Actin a-Smooth muscle-Cy3 (Sigma, Clone 1A4, #C6198)
896 product citations:https://www.sigmaaldrich.com/SE/en/search/c6198?focus=papers&page=1&perpage=30&sort=relevance&term=C6198&type=citation_search

Antibodies used for mouse PLA assay (dilution 1:200):

Rabbit anti-phospho-Tyrosine (P-Tyr-1000 MultiMabTM, Cell Signaling, #8954),
178 product citations:https://www.cellsignal.com/products/primary-antibodies/phospho-tyrosine-p-tyr-1000-multimab-rabbit-mab-mix/8954?srsltid=AfmBOop2hzNtALQP7_8jBGx2rQKEGUyBEHcjKVEdXJeOAuF2-yoqHORi

Goat anti-mouse/rat TIE2 (R&D Systems,#AF762),
37 product citations: https://www.rndsystems.com/products/mouse-rat-tie-2-antibody_af762#product-citations

Antibodies used for human PLA assay (dilution 1:200):

Rabbit anti-phospho-Tyrosine (P-Tyr-1000 MultiMabTM, Cell Signaling, #8954),
178 product citations:https://www.cellsignal.com/products/primary-antibodies/phospho-tyrosine-p-tyr-1000-multimab-rabbit-mab-mix/8954?srsltid=AfmBOop2hzNtALQP7_8jBGx2rQKEGUyBEHcjKVEdXJeOAuF2-yoqHORi

Goat anti-human TIE2 (R&D Systems, #AF313),
44 product citations:https://www.rndsystems.com/products/human-tie-2-antibody_af313#product-citations

Antibodies for pTIE2 staining (sections):

Primary:
Rabbit anti-mouse/human phospho-Tie2 (pY992) (R&D Systems, #AF2720), dilution 1:200
33 product citations: https://www.rndsystems.com/products/human-mouse-phospho-tie-2-y992-antibody_af2720#product-citations

Secondary:
Donkey anti-rabbit IgG Highly Cross-Adsorbed AF647 (#A32795), dilution 1:500
antibody testing data: https://www.thermofisher.com/antibody/product/Donkey-anti-Rabbit-IgG-H-L-Highly-Cross-Adsorbed-Secondary-Antibody-Polyclonal/A32795
283 product citations:https://www.thermofisher.com/antibody/product/Donkey-anti-Rabbit-IgG-H-L-Highly-Cross-Adsorbed-Secondary-Antibody-Polyclonal/A32795

Antibodies used for intravital imaging:

Anti-human, mouse Alexa Fluor 647-labeled, non-blocking PECAM-1 antibody (Clone 390, #102416, BioLegend), 25 µg intravenous injection
47 product citations:https://www.biolegend.com/en-gb/products/alexa-fluor-647-anti-mouse-cd31-antibody-3092

Antibodies used for ChIP-seq:

ChIP-grade rabbit antibody against human FOXO1A (abcam, #ab39670), 4 µg
94 product citations: https://www.abcam.com/en-us/products/primary-antibodies/foxo1a-antibody-ab39670?

srsltid=AfmBOopQDwHfTR9Xh7rAe-b52Tvzf21fNuCwctzTiCUJ-IRzLr0Tk4AN

ChIP-grade mouse antibody against H3K4me3 (Active Motif,#39159),4 µg
51 product citations: https://www.thermofisher.com/antibody/product/Histone-H3K4me3-Antibody-Polyclonal/39159

ChIP-grade rabbit antibody against H3K27ac (Active Motif, #39133), 4 µg
42 product citations: https://www.thermofisher.com/antibody/product/Histone-H3K27ac-Antibody-Polyclonal/39133

Antibodies Immunoprecipitation

1µg goat anti-human TIE2 (R&D Systems, #AF313),
44 product citations:https://www.rndsystems.com/products/human-tie-2-antibody_af313#product-citations

Western blot analysis

Rabbit anti-human ANGPT2 (D200) (Cell Signaling Technologies , #50697), 1:1000
https://www.cellsignal.com/products/primary-antibodies/angiopoietin-2-d200-antibody/50697
citation: DOI: 10.1155/2022/6595778

Rabbit anti-human pan AKT (Cell Signaling Technologies, #4691), 1:1000
5677 product citations: https://www.cellsignal.com/products/primary-antibodies/akt-pan-c67e7-rabbit-mab/4691

Rabbit, anti-human phospho-AKT (D9E) (Ser473) (Cell Signaling Technologies, #4060), 1:1000
11915 product citations: https://www.cellsignal.com/products/primary-antibodies/phospho-akt-ser473-d9e-xp-rabbit-mab/4060

Mouse anti-human phospho-TYR (#Cell Signaling Technologies, #96215), 1:1000
28 product citations: https://www.cellsignal.com/products/primary-antibodies/phospho-tyrosine-4g10-mouse-mab/96215

Rabbit anti-human S6 ribosomal protein (5G10) (Cell Signaling Technologies, #2217), 1:5000
2453 product citations:

Rabbit anti-human phospho-S6 ribosomal protein (91B2) (Ser235/236) (Cell Signaling Technologies, #4857), 1:5000
239 product citations: https://www.cellsignal.com/products/primary-antibodies/phospho-s6-ribosomal-protein-ser235-236-91b2-rabbit-mab/4857

Rabbit anti-human: α/β-Tubulin (Cell Signaling Technologies, #2148), 1:5000
797 product citations: https://www.cellsignal.com/products/primary-antibodies/a-b-tubulin-antibody/2148

Rabbit V5-tag (D3H8Q) (Cell Signaling Technologies, #13202), 1:2500
474 product citations: https://www.cellsignal.com/products/primary-antibodies/v5-tag-d3h8q-rabbit-mab/13202

Goat anti-human TIE2 antibody (R&D Systems, #AF313), 1:1000
44 product citations:https://www.rndsystems.com/products/human-tie-2-antibody_af313#product-citations

HRP-conjugated secondary antibodies

Goat anti-rabbit antobody (Jackson Immuno Reserach Labs, #111-035-008), 1:5000
87 product citations: https://www.jacksonimmuno.com/catalog/products/111-035-008

Rabbit anti-mouse antibody (Jackson Immuno Reserach Labs, 315-035-003) 1:5000
100 product citations: https://www.jacksonimmuno.com/catalog/products/315-035-003

Rabbit and anti-goat antibody (Jackson Immuno Reserach Labs, 305-036-008) 1:5000
2 product citations: https://www.jacksonimmuno.com/catalog/products/305-036-008

Validation

All antibodies, except for anti-ANGPT2, are commercially available and validated for the specified species and applications by the indicated manufacturer and/or in previous publications by us and others. Details are available on the manufacturers' website indicated for each antibody above.

Humanized anti-ANGPT2 antibody (ABTAA, provided by Gou Young Koh and not commercially available) was validated in: Han, S. et al. Amelioration of sepsis by TIE2 activation–induced vascular protection. Sci. Transl. Med. 8, 335ra55-335ra55 (2016).

Eukaryotic cell lines

Policy information about cell lines and Sex and Gender in Research

Cell line source(s)	Pooled human umbilical vein endothelial cells (HUVECs) were purchased from Lonza (#CC-2519). Human embryonic kidney cells (HEK293FT) were purchased from Life Technologies (R70007).
Authentication	No commonly misidentified cell lines were used. For data analysis only primary cells (HUVECs) were used, which were validated by the commercial vendor and cultured for a limited number of passages.
Mycoplasma contamination	All cells were tested negative for mycoplasma contamination.
Commonly misidentified lines (See ICLAC register)	No commonly misidentified lines were used in this study.

Animals and other research organisms

Policy information about studies involving animals; ARRIVE guidelines recommended for reporting animal research, and Sex and Gender in Research

Laboratory animals	R26-LSL-Pik3caH1047R mice (Eser et al, 2013) were crossed with the Cdh5-CreERT2 mice (Want et al, 2010) or Vegfr1-CreERT2 mice (Petkova et al. 2023), and analyzed on a C57BL/6J background. For clonal tracing, the mice were further crossed with the R26-iChr2-Mosaic animals (Pontes-Quero et al. 2017). Both female and male mice were used for analysis and no differences in the phenotype between the sexes were observed. Postnatal mice between the age of 3-16 weeks were used for experiments, and the age is stated in the figures and/or legends. Mice were housed in individually ventilated cages under a 12:12-h dark–light cycle (light from 07:00 to 19:00) at 22 ± 1°C with ad libitum access to food and water.
Wild animals	The study does not contain wild animals.
Reporting on sex	Sex was used as a biological variable in the analyses, and as reported in the manuscript, no sex-related differences were observed.
Field-collected samples	The study does not contain samples collected from the field.
Ethics oversight	Experimental procedures on mice were approved by the Uppsala Animal Experiment Ethics Board (permit numbers 130/15, 5.8.18-06383/2020, 5.8.18-03362/2021) and performed in compliance with all relevant Swedish regulations.

Note that full information on the approval of the study protocol must also be provided in the manuscript.

Plants

Seed stocks	n/a
Novel plant genotypes	n/a
Authentication	n/a

ChIP-seq

Data deposition

☒ Confirm that both raw and final processed data have been deposited in a public database such as GEO.

☒ Confirm that you have deposited or provided access to graph files (e.g. BED files) for the called peaks.

Data access links *May remain private before publication.*	Datasets generated in this study have been deposited in the Gene Expression Omnibus under accession number GSE128636.
Files in database submission	Files available in the database submission:<br><br>Processed data files<br>HUVEC_AdControl_FOXO1_ChIPseq.bw<br>HUVEC_AdFOXO1A3_FOXO1_ChIPseq.bw<br>HUVEC_AdControl_H3K27ac_ChIPseq.bw

HUVEC_AdFOXO1A3_H3K27ac_ChIPseq.bw
HUVEC_AdControl_H3K4me3_ChIPseq.bw
HUVEC_AdFOXO1A3_H3K4me3_ChIPseq.bw
HUVEC_pooled_input.bw

Raw files
HUVEC_AdControl_FOXO1_ChIPseq.fastq.gz
HUVEC_AdFOXO1A3_FOXO1_ChIPseq.fastq.gz
HUVEC_AdControl_H3K27ac_ChIPseq.fastq.gz
HUVEC_AdFOXO1A3_H3K27ac_ChIPseq.fastq.gz
HUVEC_AdControl_H3K4me3_ChIPseq.fastq.gz
HUVEC_AdFOXO1A3_H3K4me3_ChIPseq.fastq.gz
HUVEC_pooled_input.fastq.gz

Genome browser session (e.g. UCSC)

Provide a link to an anonymized genome browser session for "Initial submission" and "Revised version" documents only, to enable peer review. Write "no longer applicable" for "Final submission" documents.

Methodology

Replicates

1; Pooled chromatin from independent transductions.

Sequencing depth

Illumina sequencing libraries were prepared from the ChIP and Input DNAs by the standard consecutive enzymatic steps of end-polishing, dA-addition, and adaptor ligation.
After a final PCR amplification step, the resulting DNA libraries were quantified and sequenced on Illumina's NextSeq 500 (75 nt reads, single end).
HUVEC-AdControl_FOXO1 45053443 reads
HUVEC-AdFOXO1A3_FOXO1 47702593 reads
HUVEC-AdControl_H3K27Ac 38862996 reads
HUVEC-AdFOXO1A3_H3K27Ac 37462857 reads
HUVEC-AdControl_H3K4me3 38628403 reads

Antibodies

ChIP-grade antibodies: FOXO1 (abcam, #ab39670), H3K4me3 (Active Motif, #39159),H3K27ac (Active Motif, #39133)

Peak calling parameters

Peak locations were determined using the MACS algorithm (v2.1.0) with a cutoff of p-value = 1e-7. Peaks that were on the ENCODE blacklist of known false ChIP-Seq peaks were removed.

Data quality

Data quality was assessed with the FastQC quality-control tool for high throughput sequence data.

Software

MACS, BWA (v0.7.12), bcl2fastq2 (v2.20), Samtools (v0.1.19), BEDtools (v2.25.0), wigToBigWig (v4)
