# Supplementary figures and images for: Angiopoietin–TIE2 feedforward circuit promotes PIK3CA-driven venous malformations
Source: Nat Cardiovasc Res. 2025 May 23;4(7):801–20. doi: 10.1038/s44161-025-00655-9 (PMC12259471; doi:10.1038/s44161-025-00655-9)

Figure 5e

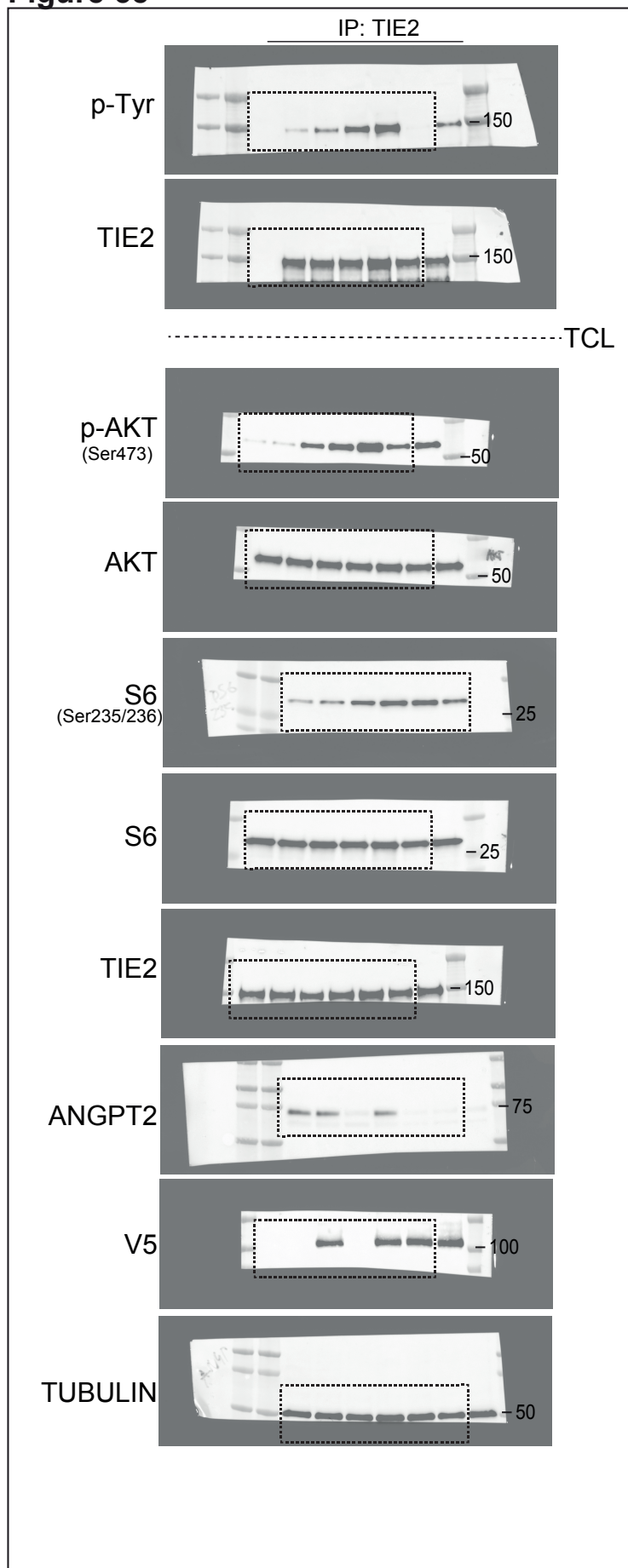

Figure 5f

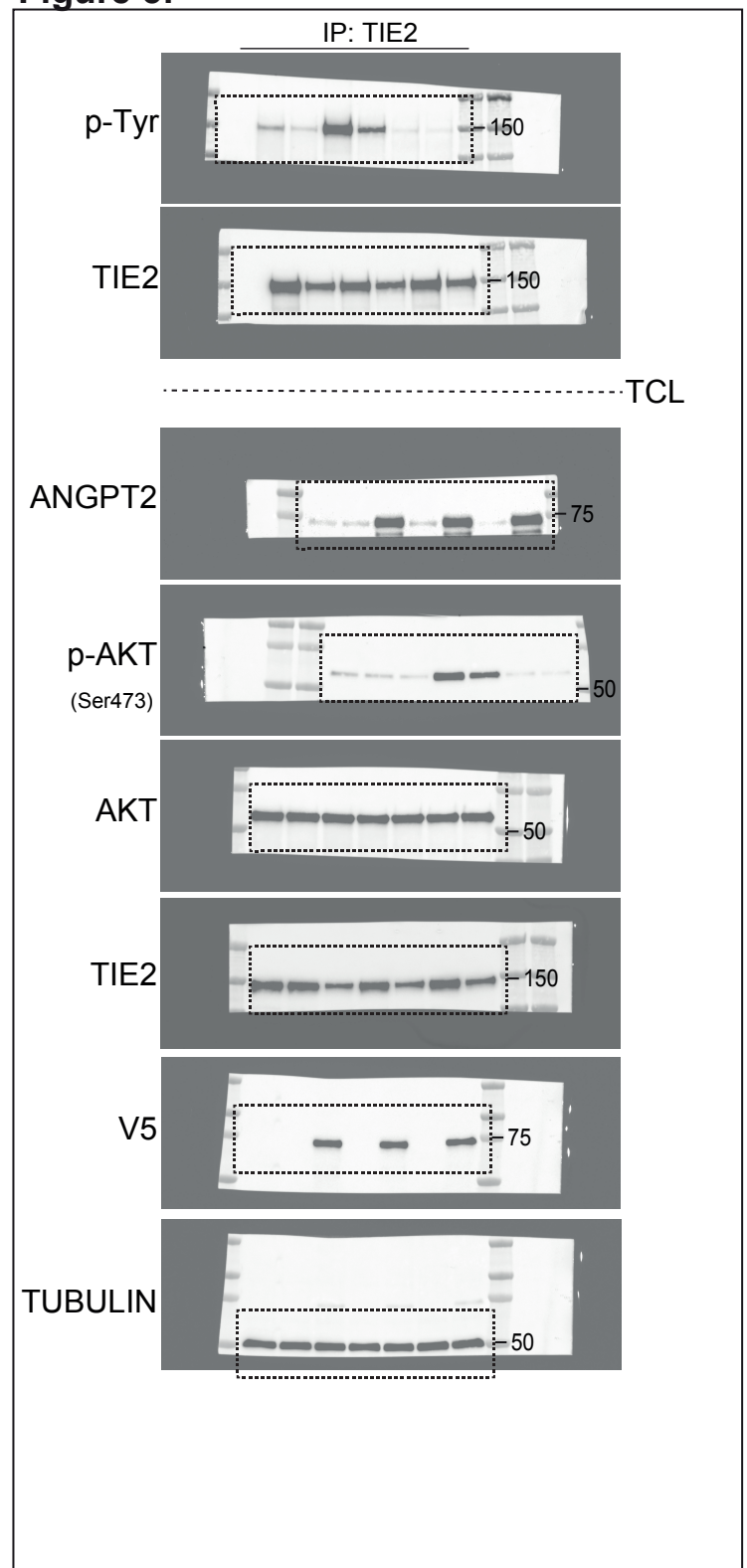

Supplement: Supplementary file 18 — Unprocessed western blots for Fig. 5e,f. [file 44161_2025_655_MOESM18_ESM.pdf]
